# A CIN-like TCP transcription factor (*LsTCP4*) having retrotransposon insertion associates with a shift from Salinas type to Empire type in crisphead lettuce (*Lactuca sativa* L.)

**DOI:** 10.1038/s41438-020-0241-4

**Published:** 2020-02-01

**Authors:** Kousuke Seki, Kenji Komatsu, Keisuke Tanaka, Masahiro Hiraga, Hiromi Kajiya-Kanegae, Hideo Matsumura, Yuichi Uno

**Affiliations:** 1 Nagano Vegetable and Ornamental Crops Experiment Station, Tokoo 1066-1, Souga, Shiojiri, Nagano, 399-6461 Japan; 2 Department of Bioresource Development, Tokyo University of Agriculture, Funako, 1737, Atsugi, Kanagawa, 243-0034 Japan; 3 NODAI Genome Research Center, Tokyo University of Agriculture, Sakuragaoka 1-1-1, Setagaya, Tokyo, 156-8502 Japan; 4 Nagano Fruit Tree Experiment Station, Ogawara 492, Suzaka, Nagano, 382-0072 Japan; 5 Research Center for Agricultural Information Technology, NARO, Kannondai 3-1-1, Tsukuba, Ibaraki, 305-8517 Japan; 6 Gene Research Center, Shinshu University, Tsuneta 3-15-1, Ueda, Nagano, 386-8567 Japan; 7 Plant Science Division, Department of Bioresource Science, Graduate School of Agricultural Science, Kobe University, Rokkodai 1-1, Nada, Kobe, Hyogo, 657-8501 Japan

**Keywords:** Plant breeding, Agricultural genetics

## Abstract

To improve several agronomic traits in crisphead lettuce (*Lactuca sativa* L.) under high-temperature growth conditions, we investigated the correlation among those traits in multiple cultivars and performed genetic mapping of their causal genes. In a field cultivation test of Empire type (serrated leaf) and Salinas type (wavy leaf) cultivars, Empire type cultivars showed increased tipburn susceptibility and late bolting compared with Salinas type cultivars. We attempted genetic mapping of leaf shape and bolting time by ddRAD-seq using the F_2_ population derived from a cross between ‘VI185’ (Empire type) and ‘ShinanoGreen’ (Salinas type). These analyses suggested that both traits are controlled by a single locus in LG5. Genotyping of 51 commercial lettuce cultivars with a tightly linked marker (*LG5_v8_252.743Mbp*) at this locus showed an association between its genotype and the serrated leaf phenotype. By further fine mapping and transcriptome analysis, a gene encoding putative CIN-like TCP transcription factor was determined to be a candidate gene at this locus and was designated as *LsTCP4*. An insertion of retrotransposable element was found in the allele of ‘VI185’, and its transcript level in the leaves was lower than that in ‘ShinanoGreen’. Because shapes of leaf epidermal cells in ‘VI185’ were similar to those in the TCP family mutant of *Arabidopsis thaliana*, the leaf shape phenotype was likely caused by reduced expression of *LsTCP4*. Furthermore, because it is known that the TCP family protein also controls flowering time via interaction with FT in *A. thaliana*, it was highly possible that *LsTCP4* gave pleiotropic effects on both leaf shape and bolting time in lettuce.

## Introduction

**Fig. 1:**
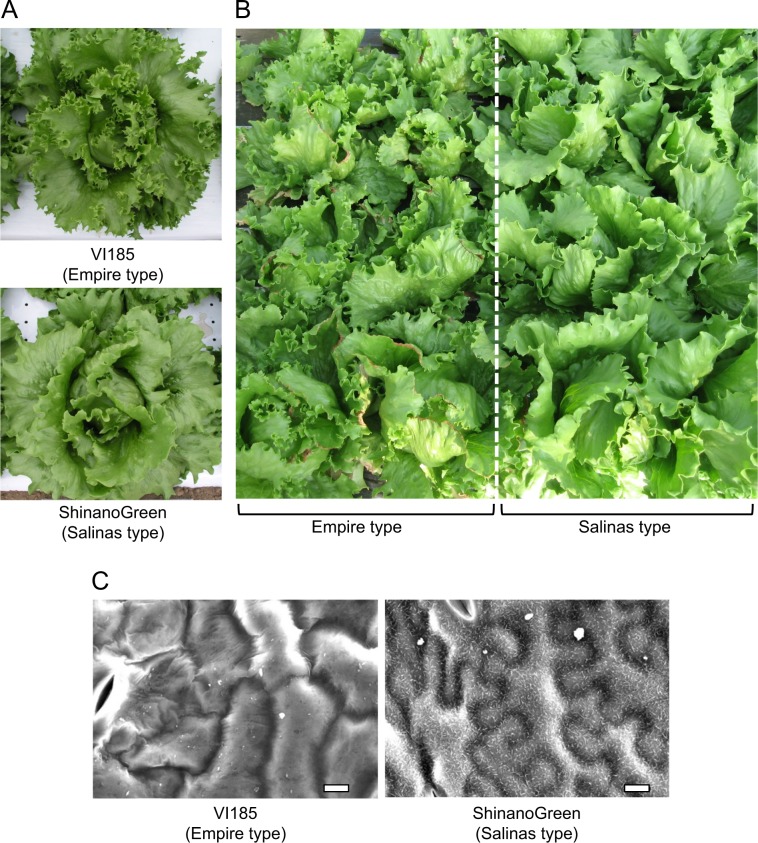
Features of two types of crisphead lettuce. **a** The undulation shape of leaf margin differs between ‘VI185’ (Empire type) and ‘ShinanoGreen’ (Salinas type) of crisphead lettuce. Empire type has serrated leaf margin, while Salinas type has wavy leaf. **b** Empire type tend to be more susceptible to tipburn than Salinas type. **c** Paradermal view of epidermal cells in Empire type and Salinas type using SEM. Bar = 100 µm

In the field, how does the leaf shape impact on the agronomic characterization of cultivars? Because the leaf shape of leafy vegetables is a very important agronomic trait, it has been diversified by breeders worldwide. The diversity of cultivars plays a key role in adaptation to stressful cultivation environments. Lettuce (*Lactuca sativa* L.) is the most popular leafy vegetable in the world^1^. It is cultivated throughout the year and is traditionally grown outdoors, with the exception of butterhead lettuce^2^. For cool-season vegetables such as lettuce, environmental factors, particularly heat stress, can have negative effects on yield and quality. Accordingly, for stable production during warmer seasons, temperature adaptability is a critical trait for lettuce cultivars. Crisphead lettuce has two major groups of cultivars, known as the Empire type and Salinas type^3,4^. The Empire type exhibits deeply serrated leaf margins with a crisp texture, and the Salinas type has wavy leaves with a softer texture (Fig. 1a). The serrated leaf margin in the Empire type is clearly associated with the high incidence of tipburn (Fig. 1b)^4,5^.

Tipburn is a complex and unpredictable physiological disorder that involves the collapse and necrosis of the apex and margins in actively growing leaves. It is one of the most critical problems in lettuce cultivation under warm growing conditions, where tipburn occurs at high frequencies. Bolting resistance is also an important trait in lettuce growth in summer. ‘Salinas’ (alias iceberg saladin) is regarded as a late-bolting cultivar in the United States^6^. However, under high-temperature cultivation conditions, such as those found in midsummer in Japan, ‘Salinas’ is categorized as an early-bolting type. In midsummer cultivation, the period from seeding to harvest lasts 70–80 days in the United States^7^, whereas in Japan, it lasts 50–65 days at 30 °C. Jenni et al. also pointed out that the Salinas type cultivars exhibited early bolting (longer stems) under long days and warm growing conditions^4^. Because bolting of lettuce is generally promoted by high temperatures^8^ and leaves of bolted plants are deemed to be unsaleable due to their bitterness^7^, bolting resistance is an important trait under higher temperatures. During the high temperatures in midsummer and in subtropical regions, the improvement in resistance to tipburn and bolting has been an important goal of lettuce breeding.

In the present study, we investigated the correlations among leaf shape, tipburn, and bolting in multiple lettuce cultivars under hot cultivation environments. In addition, genetic mapping of leaf shape and late-bolting phenotypes was performed in the F_2_ population derived from Empire type and Salinas type cultivars to elucidate the genetic relationships between the two traits. In these studies, a causal candidate gene and widely available marker for these traits were identified using multiple NGS-based tools.

## Results

### Phenotypic evaluation of lettuce cultivars in the field

To evaluate phenotypic differences between the two types of crisphead lettuce, five Empire type and five Salinas type cultivars (10 cultivars total) were cultivated in the field for 5 years (2013–2016 and 2018). All plants were grown during the midsummer season (June–August) in Japan. All the Empire type cultivars exhibited serrated leaves with undulation, whereas Salinas type cultivars exhibited wavy leaves without serrated leaf margins (Fig. 1), as described previously^4^. Stem length as the standard of bolting time as well as tipburn occurrence were scored in individual plants. Empire type cultivars had shorter stems compared with Salinas types, whereas the standard deviations in Salinas type cultivars were large (Table 1, Table S1).

**Table 1:** Difference in bolting times in lettuce cultivars between Empire type and Salinas type evaluated by the mean values of stem length inside the head in field cultivation tests

Year	Type	Stem length (cm)	SD	*P*-value
2013	Empire	6.03	±0.97	7.20 × 10^−3^
	Salinas	7.62	±2.9	
2014	Empire	6.80	±1.47	0.9466
	Salinas	6.78	±1.17	
2015	Empire	6.05	±1.03	0.2913
	Salinas	6.40	±1.52	
2016	Empire	8.73	±2.19	0.08999
	Salinas	10.23	±4.2	
2018	Empire	6.44	±1.69	1.76 × 10^−4^
	Salinas	11.58	±6.44	
Total	Empire	6.81	±1.82	1.29 × 10^−5^
	Salinas	8.48	±4.23	

The stem length of the Empire type was ~< 8 cm as the maximum limit of salable lettuces, suggesting late-bolting characteristics even in midsummer in Japan (Table 1, Table S1). Although tipburn did not always uniformly occur every year, its frequency in Empire type cultivars was higher than that in the Salinas type. It was more likely to be observed in inner leaves than in outer ones in both types (Table 2, Table S2).

**Table 2:** The difference in tipburn susceptibility in lettuce cultivars between Empire type and Salinas type evaluated by both inside and outside the head in field cultivation tests

Year	Type	Outside tipburn				Inside tipburn			
		%Tipburn	No. with tipburn	No. no tipburn	*P*-value	%Tipburn	No. with tipburn	No. no tipburn	*P*-value
2013	Empire	34.0	51	99	1.43 × 10^−4^	76	38	12	3.22 × 10^−7^
	Salinas	14.7	22	128		24	12	38	
2014	Empire	8.1	13	148	0.3658	78	39	11	6.82 × 10^−6^
	Salinas	5.2	8	145		32	16	34	
2015	Empire	23.1	40	133	9.00 × 10^−14^	100	50	0	1.13 × 10^−10^
	Salinas	0.0	0	150		46	23	27	
2016	Empire	20.0	30	120	1.91 × 10^−4^	100	50	0	9.98 × 10^−5^
	Salinas	5.3	8	142		74	37	13	
2018	Empire	59.3	89	61	<2.2 × 10^−16^	84	42	8	2.04 × 10^−7^
	Salinas	12.0	18	132		32	16	34	
Total	Empire	28.4	223	561	<2.2 × 10^−16^	64.6	219	120	<2.2 × 10^−16^
	Salinas	7.4	56	697		36.1	104	184	

These results suggest that leaf shape in the Empire and Salinas types is correlated with two important agronomic traits, bolting time and tipburn occurrence, among the cultivars used in the present study.

### Inheritance of leaf marginal serration and bolting

To understand the inheritance of leaf shape and bolting time in these two types of crisphead lettuce, an F_2_ population was developed from a cross between ‘VI185’ (Empire type) and ‘ShinanoGreen’ (Salinas type), and their phenotypes were scored. The F_1_ plants consistently showed a wavy leaf phenotype (Salinas type phenotype), suggesting its dominance. Of the 96 F_2_ individuals, 33 plants showed a serrated leaf margin phenotype, and a wavy leaf phenotype was observed in the remaining 63 plants. In addition, the F_3_ progeny from each F_2_ individual were grown, and their leaf phenotype was also evaluated. All the F_3_ progenies derived from F_2_ plants with a serrated leaf margin uniformly showed the same leaf phenotype as their parent, indicating that these F_2_ plants were homozygotes of the allele responsible for serrated leaf phenotype. In the F_2_ plants showing a wavy leaf phenotype, all the F_3_ progenies of 19 plants showed a leaf phenotype similar to that of their parents, whereas leaf phenotypes were segregated in the progenies of 44 F_2_ plants (Table 3). This suggests that leaf shape (serrated leaf margin or wavy leaf) is determined by a single locus and the wavy leaf phenotype is dominant.

**Table 3:** *χ*^2^ test for segregation of leaf shape in F_2_ populations derived from ‘VI185’ and ‘ShinanoGreen’

Population	Total	Serrated leaf	Wavy leaf (Hetero)	Wavy leaf (Homo)	Segregation ratio	*χ*^2^ (1:2:1)
VI185	10	10	–	0	–	–
ShinanoGreen	10	0	–	10	–	–
F_2_	96	33	44	19	1:1.3:0.6	0.09

**Fig. 2:**
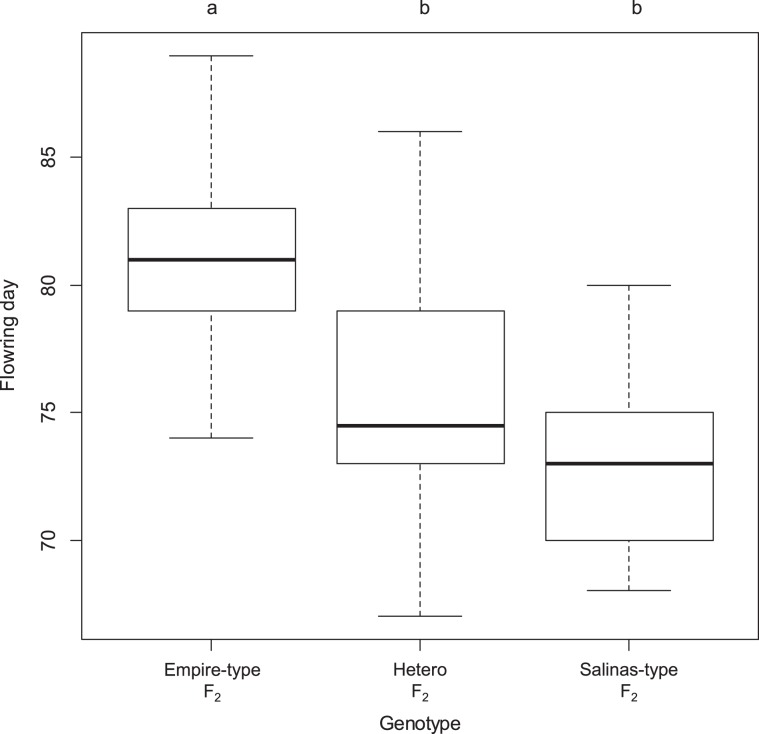
The difference in bolting tolerance among three genotypes of putative leaf shape locus. The bolting tolerance was evaluated by the days to flowering. Different alphabet above boxplot represents significant difference (*P* < 0.01) among three genotypes

The flowering day of each F_2_ plant was also recorded as the phenotype of bolting time. Scores of the flowering day were apparently segregated in accordance with genotypes of leaf shape locus, as described above. F_2_ plants with serrated leaf margins showed later flowering days as compared with wavy leaf plants. In the wavy leaf F_2_ plants, a significant difference in bolting time was observed between putative homozygotes and heterozygotes at the putative leaf shape locus, which were deduced from the segregation in F_3_ (Fig. 2).

These results suggest that leaf shape and flowering days (bolting time) are genetically controlled by the same locus or closely linked loci, and its heterozygote probably has a semi-dominant effect on bolting time.

### ddRAD-seq analysis of parental lines

**Fig. 3:**
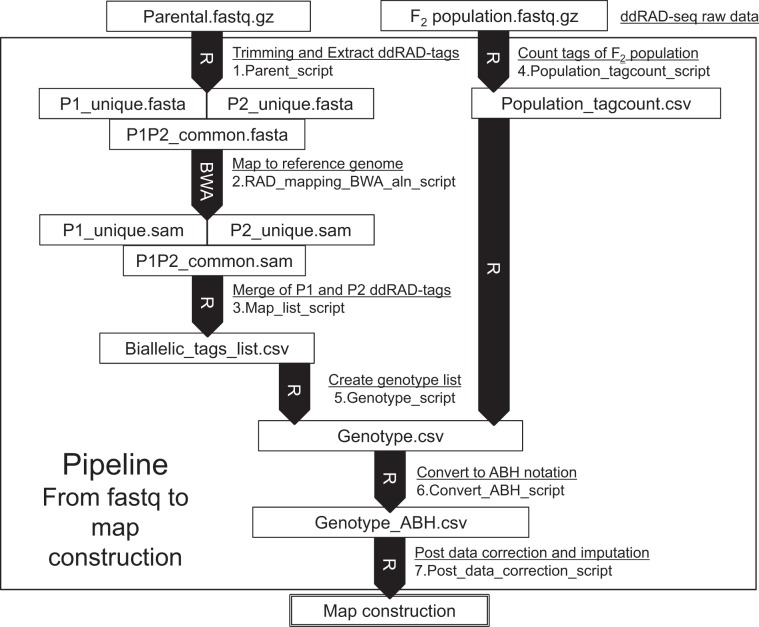
Pipeline workflow of ddRAD-seq data analysis from sequence reads to linkage map

For genetic mapping of the locus for serrated leaf margin and late bolting observed in the Empire type, ddRAD-seq analysis was conducted for molecular marker development and its genotyping in the F_2_ population (Fig. 3).

First, the genomic DNA polymorphisms between two parental lines, ‘VI185’ (Empire type) and ‘ShinanoGreen’ (Salinas type), were assessed by ddRAD-seq analysis using PacI and NlaIII as restriction enzymes. From Illumina HiSeq sequencing of ddRAD-seq libraries, 6,031,184 and 6,279,218 single reads (100 bp) were obtained from ‘VI185’ and ‘ShinanoGreen’ plants, respectively. RAD tags were extracted from the sequence reads for individual samples. In total, 366,846 and 364,689 RAD tags showing more than two count reads were obtained in the ‘VI185’ and ‘ShinanoGreen’ samples, respectively. By comparing the RAD tags of two parental lines, 78,587 and 75,489 unique tags were identified as ‘VI185’ or ‘ShinanoGreen’-specific tags, respectively, whereas 242,106 RAD tags commonly appeared in both samples. The read mapping was performed with these unique RAD tags in each parent against the lettuce reference genome sequence [version8 from crisphead cultivar ‘Salinas’ (https://genomevolution.org/coge/GenomeInfo.pl?gid=28333)]. The 4992 pairs of RAD tags (designated as biallelic tags) harboring SNPs or InDels in two parental lines were defined (Table S3), and these biallelic tags were employed as co-dominant markers for further genetic mapping.

### ddRAD-seq analysis of the F_2_ population and linkage map development

Genotypes of these 4992 biallelic tag loci in the F_2_ population of a cross between ‘VI185’ and ‘ShinanoGreen’ were also determined by the ddRAD-seq analysis of 96 F_2_ individuals. Genotypes of biallelic tag loci in 96 F_2_ individuals were determined based on the presence or absence of each allelic tag. After excluding loci showing many missing RAD tag data, genotyping data on 4517 biallelic tag loci in 96 F_2_ individuals were used for linkage map construction (Fig. S1). By summarizing co-segregated biallelic tag loci, 840 loci were regarded as the co-dominant markers (Table S3), showing different segregations in 96 F_2_ individuals. Using grouping analysis, all the analyzed marker loci were distributed into nine linkage groups, and by ordering the marker loci in each linkage group, a linkage map encompassing 1529.2 cM was newly developed. Summary statistics of the linkage map are shown in Table S3. Marker density ranged from 0.2 cM per marker (LG1, LG2, LG5) to 1.5 cM per marker (LG4). The number of markers ranged from 140 (LG4) to 810 (LG5).

### Fine mapping of leaf marginal serration locus and candidate gene analysis

By quantitative trait loci (QTL) detection with composite interval mapping (CIM) of the serrated leaf margin phenotype using genotype data from the ddRAD-tag data of 96 F_2_ individuals, we found that a single locus for leaf shape was located in LG5, flanked by two markers (*LG5_v8_244.527Mbp* and *LG5_v8_256.311Mbp*) at a 6.22 cM interval. Similarly, a locus for flowering day was found to be located in the same region as the leaf shape locus (Table 4).

**Table 4:** Positions of locus for leaf shape and flowering day in the linkage map and their genetic effects

Trait name	Linkage group	Marker	Genetic distance (cM)	Physical position (cM)	Threshold LOD	LOD	Additive effect	Dominant effect	*R*^2^ (%)
Leaf marginal serration	LG5	LG5_v8_244.527Mbp to LG5_v8_256.311Mbp	6.22	130.78–137.00	4.82	131.36	50	−25.54	99.79
Flowering Day	LG5	LG5_v8_244.527Mbp to LG5_v8_256.311Mbp	6.22	130.78–137.00	4.67	6.39	4.65	−1.41	28.68

Because leaf shape (serrated leaf margin phenotype) was apparently determined by the strong effect of a single locus according to the CIM result, we tried to pinpoint the position of its locus further based on the genotypes of biallelic RAD tags around this region and phenotypic segregation in F_2,3_ plants as shown (Table 3). This demonstrated that the locus of leaf marginal serration was located from 251.386 to 253.367 Mbp at an interval of 2.1 cM on LG5, and the genotype of the marker designated as LG5_v8_252.185 Mbp showed complete co-segregation with leaf phenotype based on the present F_2_ population. Moreover, fine mapping of the target locus was performed using six markers (Table S4). Consequently, we succeeded in narrowing down the locus for leaf marginal shape to within a 1.33 Mbp region and found that four markers located in this region (*LG5_v8_251.738Mbp*, *LG5_v8_252.704Mbp_DdeI*, *LG5_v8_252.743Mbp*, and *LG5_v8_252.927Mbp*) showed tight linkage to the serrated leaf margin phenotype in the analyzed F_2_ individuals (Table S4).

**Fig. 4:**
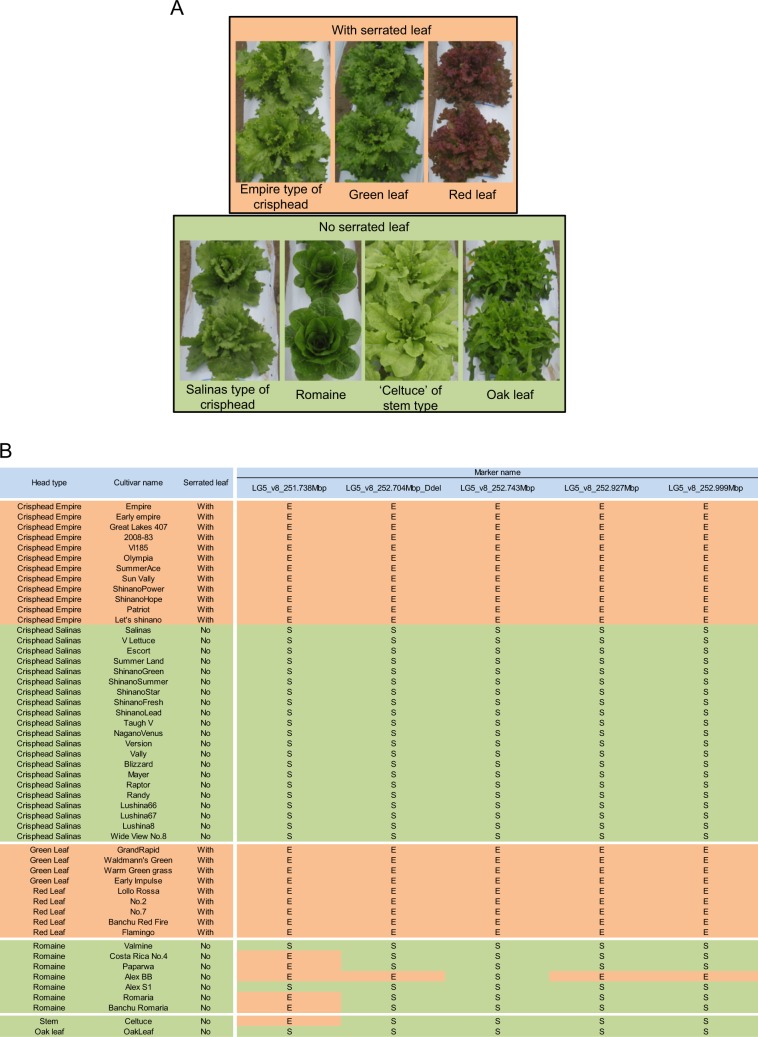
Leaf shape and genotype of multiple markers in various lettuce cultivars. **a** Leaf marginal shape for each lettuce type was separated into “With serrated leaf” or “No serrated leaf”. **b** Phenotype of leaf marginal shape, and genotype using 6 markers were compared in 51 cultivars. ‘Empire’ indicates the same genotype as Crisphead’s ‘Empire’ cultivar, ‘Salinas’ indicates the same genotype as Crisphead ‘Salinas’ cultivar

In addition, 51 cultivars classified as five horticultural types of lettuce were also used for genotyping of these six markers to evaluate their genetic association (Fig. 4a, b).

**Fig. 5:**
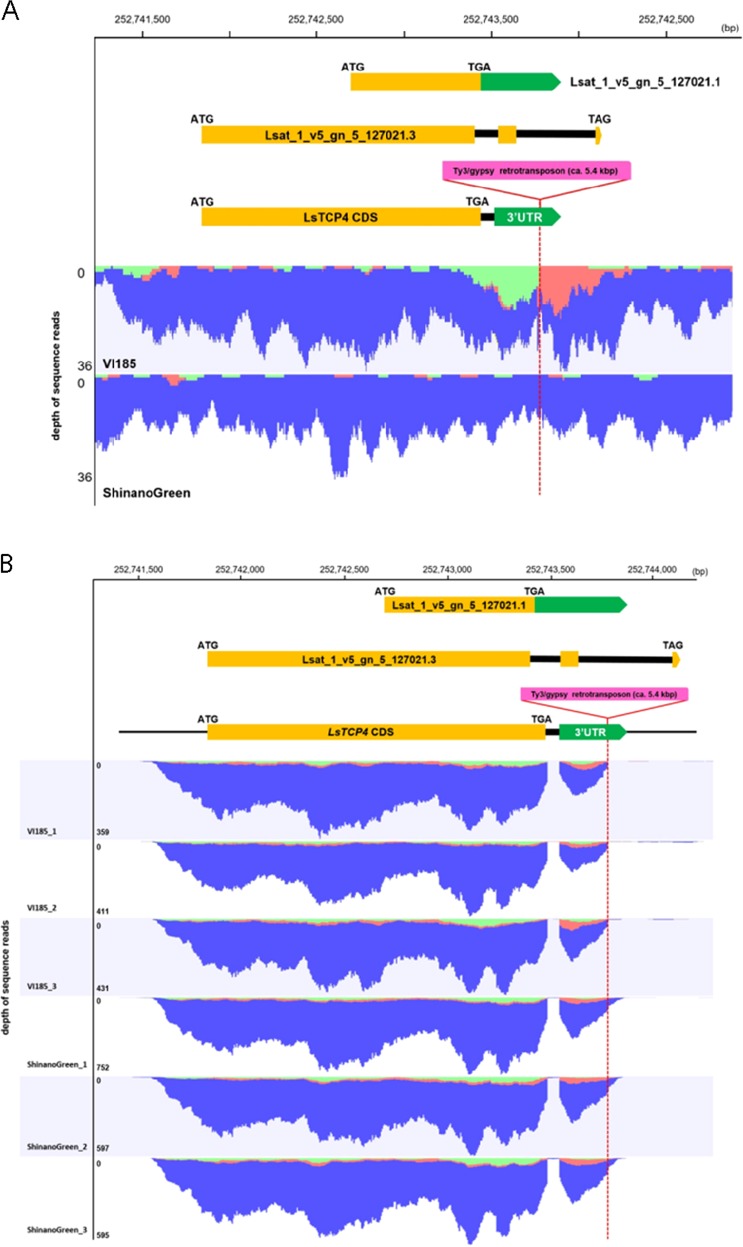
Comparison of genomic sequences of Lsat_1_v5_gn_5_127021 locus in ‘VI185’ (Empire type) and ‘ShinanoGreen’ (Salinas type). **a** Gene model of *LsTCP4* and mapped genomic DNA-seq reads in Lsat_1_v5_gn_5_127021 locus. Paired-end reads from ‘VI185’ and ‘ShinanoGreen’ were mapped against *L. sativa* V8 reference genome. Reads were distinguished by blue (pair-mapped reads), green (forward reads as single-mapped), and red (reverse reads as single-mapped). Gene models in Lsat_1_v5_gn_5_127021 loci (Lsat_1_v5_gn_5_127021.1 and Lsat_1_v5_gn_5_127021.3) and LsTCP4 were shown. Orange, green, and pink colored boxes indicated coding sequences (CDS), 3′ untranslated region (UTR) and Ty3/gypsy retrotransposon, respectively. Red dotted line indicates insertion position of Ty3/gypsy retrotransposon. **b** RNA-seq reads mapping in Lsat_1_v5_gn_5_127021 locus. Paired-end RNA-seq reads from ‘VI185’ and ‘ShinanoGreen’ leaves were mapped against *L. sativa* V8 reference sequence. Colors of mapped reads, boxes in gene model and dotted lines indicated as the same as those in panel (**a**)

Of these, 21 cultivars showed serrated marginal leaves, and the rest had Salinas type wavy leaves or other types of leaves. According to a genotype of all the cultivars, only the *LG5_v8_252.743Mbp* marker showed an association with the phenotype for leaf marginal serration (Fig. 4b, Fig. S2). Thus, it was predicted that the causal gene was located between 252.704 and 252.927 Mbp in LG5. Three candidate genes were positioned in this region, according to the reference genome sequence of *L. sativa* V8 (Table S4). Next, sequences in these three genes were compared in ‘ShinanoGreen’ and ‘VI185’. Although there were no small insertions/deletions or nonsynonymous substitutions in these three genes between the two parental lines, a putative long insertion was found at the Lsat_1_v5_gn_5_127021 locus (Fig. 5a, Table 5).

**Table 5:** Expression analysis of candidate genes in the genomic region between LG5 - 252704510 and LG5 - 252927862 in the leaves of parental lines by RNA-seq

Gene ID	Position at LG5 of reference genome sequence		*P*-value	ShinanoGreen		VI185		Putative function
	Start	End		Total reads	RPKM	Total reads	RPKM	
Lsat_1_v5_gn_5_127021	252742702	252743879	2.92 × 10^−3^	1624	57.39	932.3	34.76	TCP family transcription factor 4, putative
Lsat_1_v5_gn_5_127001	252783661	252786449	–	0	0	0	0	Expressed protein
Lsat_1_v5_gn_5_126960	252857424	252857690	–	0	0	0	0	Expressed protein

In addition, no transcripts of the Lsat_1_v5_gn_5_127001 or Lsat_1_v5_gn_5_126960 loci were detected in the RNA-seq analysis of the leaves (Table 5). Alternatively, transcripts of the Lsat_1_v5_gn_5_127021 locus were observed. These results suggest that the Lsat_1_v5_gn_5_127021 locus is involved in leaf margin serration.

**Fig. 6:**
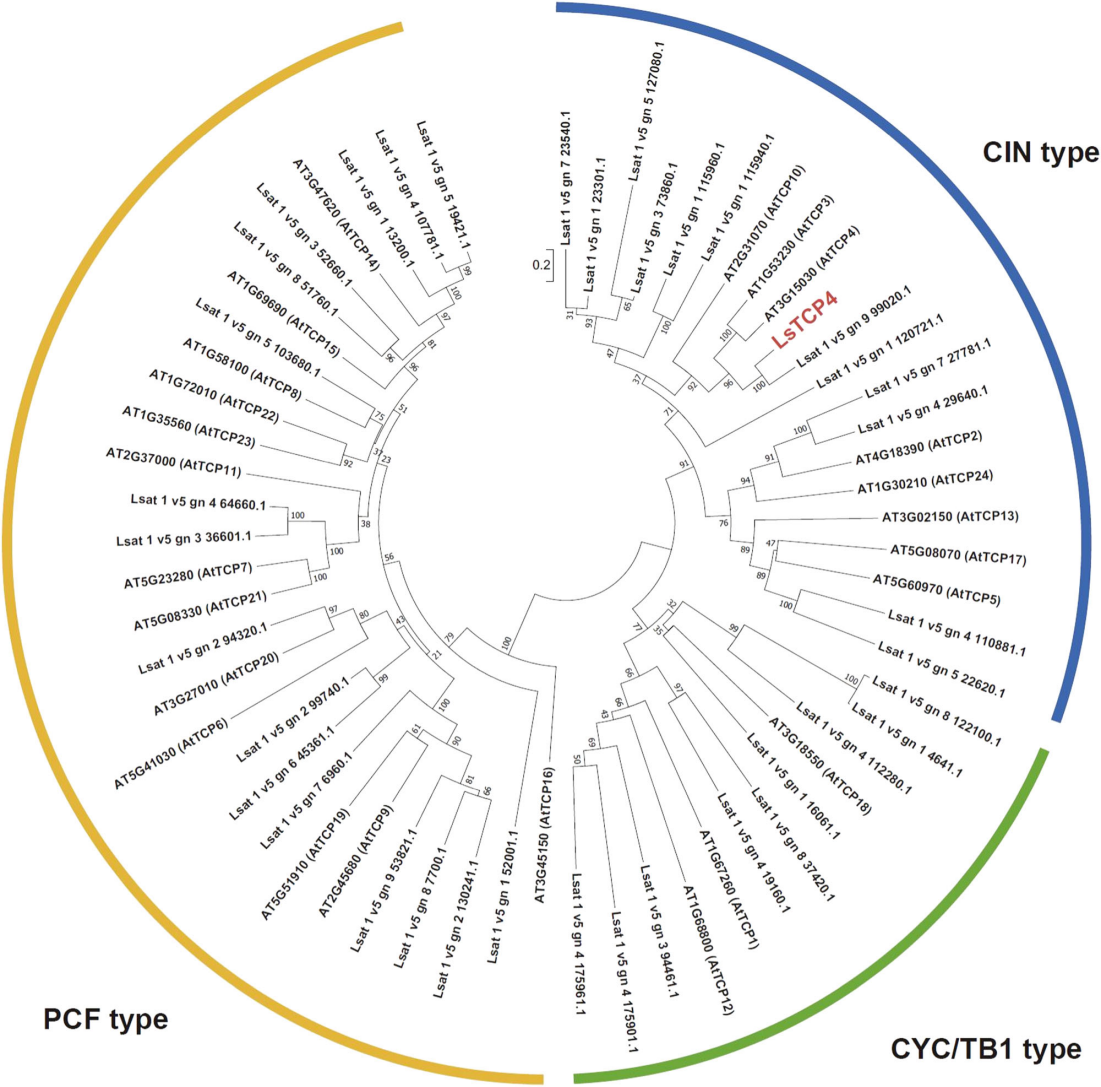
Phylogenetic analysis of TCP like proteins in *L. sativa*. Amino acid sequences of Arabidopsis and TCP family proteins and *L. sativa* TCP domain containing protein from Phytozome v12 (https://phytozome.jgi.doe.gov/pz/portal.html) were used for phylogenetic analysis. The sequences were aligned with MUSCLE. Evolutionary analyses were conducted in MEGA7 (https://www.megasoftware.net/). A neighbor-joining tree was constructed based on alignment of the amino acid sequences. The evolutionary distances were computed using the JTT matrix-based method. Numbers on branches indicate bootstrap values from 100 replicates. The bar represents number of amino acid changes per branch length

According to the annotation of the reference genome, two gene models were predicted at the Lsat_1_v5_gn_5_127021 locus. However, the two gene models did not correspond to the RNA-seq read mapping results. Thus, based on the mapping results of the RNA-seq reads, the most plausible gene model at the Lsat_1_v5_gn_5_127021 locus was reconstructed and designated as *LsTCP4* (Fig. 5a, b). Phylogenetic analysis showed that LsTCP4 was classified as the CINCINNATA (CIN)-like group of TCP family transcription factor, which is known to be related to serrated leaf margins in Arabidopsis^9–11^ (Fig. 6).

LsTCP4 has a complete TCP domain and is most similar to AtTCP4 in the Arabidopsis CIN-type TCP family (Fig. 6, Fig. S3). The de novo assembly of the ‘VI185’ genome and RNA-seq analysis showed that 5411 bp of the Ty3/gypsy retrotransposon-like sequence (Fig. S4, Fig. S5) was inserted into the 3ʹ-UTR of LsTCP4, and few RNA-seq reads corresponding to the downstream of the retrotransposon insertion were detected (Fig. 5a, b). Furthermore, the expression level of *LsTCP4* in ‘VI185’ was lower than that in ‘ShinanoGreen’, according to the RPKM in RNA-seq and quantitative RT-PCR analysis (Table 5, Fig. 5b, Fig. S6). To facilitate the detection of genetic polymorphisms, a multiplexed polymerase chain reaction (PCR) with type-specific forward primers was designed to perform simultaneous amplifications in a single reaction. The *LG5_v8_252.743Mbp_Empire_F* primer designed for the ‘VI185’-specific sequence only corresponded to retrotransposon insertion (Fig. S5). The size of the PCR products of the marker and positional relationship of the primers matched the sequence obtained by de novo assembly analysis (Fig. S2, Fig. S5). These results suggest that retrotransposon insertion into *LsTCP4* is an Empire type cultivar-specific genomic structural variant.

### Observation of the leaf surface

The leaf surfaces of ‘ShinanoGreen’ and ‘VI185’ were observed using scanning electron microscope (SEM) to compare the array patterns and shapes of their epidermal cells, which are known to be the characteristics of TCP mutants in Arabidopsis. The epidermal cells of ‘ShinanoGreen’ leaves exhibited a pavement-like pattern array, whereas those of ‘VI185’ showed exhibited a rounded shape (Fig. 1c).

The rounded shape of the leaf epidermal cells closely resembled that in the TCP family mutant of Arabidopsis^9^. Particulate epicuticular wax could only be observed on the leaf surface of ‘ShinanoGreen’.

## Discussion

In this study, we proposed that leaf marginal serration in Empire type is due to reduced amount of the *LsTCP4* transcript owing to retrotransposon insertion in the 3ʹ-UTR (Figs. 5a, b, 6, Table 5). It was reported that long 3′-UTR promoted mRNA instability in higher plants^12^. So, we presumed that long 3′-UTR with retrotransposon insertion in Empire type caused unstable mRNA. Because the Empire type showed rounded leaf epidermal cells, as observed in TCP family mutants in *Arabidopsis* (Fig. 1c), this also supported the correlation between *LsTCP4* expression and leaf shape in lettuce. TCP transcription factors are classified into two subclasses: class I and class II^13^. The class II are further separated into two subgroups: the CIN-like group and CYC/TB1 group^14^. According to the sequence similarity, *LsTCP4* are categorized into the CIN-like TCP group (Fig. 6). The CIN-like TCP transcription factors are involved in several aspects of plant development by miRNA regulation, which have been shown to influence leaf size and shape in many plants^9–11^. Eight genes were known in the CIN-like TCP group in *Arabidopsis*, and functional complementation was suggested to be present among these multiple genes. Thus, a mutation in only a single CIN-like *TCP* gene does not cause visible phenotypic change in leaves. However, present study showed that a transposon-insertion in *LsTCP4*, reducing its transcript level, altered leaf phenotype in lettuce. This was possibly due that there were structural or regulatory differences in the CIN-like *TCP* genes between *Arabidopsis* and lettuce. In addition, in *Arabidopsis*, five genes of the CIN-like TCP group are known to be targeted by miR319^15^, while in crisphead lettuce, putative miR319 target site were found in only two genes in the CIN-like TCP group (Fig. 6, Table S5), implying the difference of their transcriptional regulation. Although a paralogous gene to *LsTCP4* was found as *Lsat_1_v5_gn_9_99020.1* in the lettuce genome, its expression level was low in both ‘ShinanoGreen’ and ‘VI185’ (Table S5). Therefore, it was not expected that this paralogous gene could complement reduced expression of *LsTCP4* in Empire type lettuce. High levels of miR319 or low TCP activity result in excessive cell proliferation that generates a crinkled simple leaf in *Arabidopsis*^16^. Using GUS reporter constructs, extended mitotic activity in leaf margins in the TCP mutants that have leaf marginal serration in *Arabidopsis* has also been confirmed^17^. Elucidation of the effect of the decreased expression level of *LsTCP4* is a future concern, but an excess of undulation induced in the leaf margin of the Empire type is probably the cause of tipburn under high-temperature conditions. It has been suggested that the major QTL for tipburn in LG5 has pleiotropic effects among multiple agricultural traits^4,5^. In addition, TCP family proteins in *Arabidopsis* interact with the FLOWERING LOCUS T protein^18,19^, which affects the flowering time^19^. Thus, we infer that a mutation in *LsTCP4* introduced pleiotropic effects on both leaf shape and bolting in the Empire type of crisphead lettuce.

According to Rider^2^, the earliest recorded formal public lettuce breeding program was initiated in California in 1923. Thus, the history of crisphead lettuce breeding spans only ~100 years. Phylogenetic analysis of the crisphead lettuce cultivars, including several breeding lines, has shown that bolting-resistant lines are derived from green leaf lettuce cultivar “Grand Rapid”^20^ Serrated leaf margins in Empire type lettuce are believed to be derived from green leaf lettuce. This prediction was supported by our finding that the marker genotypes linked to serrated leaf margin in LG5 were consistent in the analyzed leaf lettuce cultivars (Fig. 4b). This allele for serrated leaf margin might have been employed to introduce late bolting and serrated crisp texture into new crisphead cultivars by breeders.

According to the phenotypes in multiple cultivars under midsummer conditions in Japan, the Empire types are likely to be tipburn susceptible, whereas they showed late-bolting phenotype under high-temperature conditions (Table 1, Table S1). In addition, a correlation between serrated leaf margin and late bolting was demonstrated in the F_2_ population.

We identified a locus between 244.527 and 256.311 Mbp in LG5 for serrated leaf margin and late bolting (Table 4), and its recessive allele probably caused both phenotypes. Our results were consistent with the finding of Ryder that the traits of serration on the leaf margin are recessive. Furthermore, Jenni et al. reported that a marker designated as *AVJT-OP4* (alias CLS_S3_Contig10103) in LG5 showed a linkage with the crisphead types of lettuce and with tipburn susceptibility in the Empire type. *AVJT-OP4* was mapped at 253.685 Mbp in LG5 (data not shown) by a local BLAST program on the reference genome *Lactuca sativa* cv. ‘Salinas’ V8; thus, the locus in this paper is consistent with the position reported by Jenni et al. (Table 3, Table 4). Recently, Macias-González et al. found that the loci for leaf shape phenotype in cv. “Emperor” and cv. “Calicel” were located between Lsat_1_v5_g_5_892 (244 Mbp) and Lsat_1_v5_g_5_1614 (269 Mbp) in LG5. Thus, the identified marker *LG5_v8_252.743Mbp* could be used to distinguish almost all the serrated leaf margins in lettuce. Thus, this study revealed an important gene for improving lettuce production under stressful environments, such as high temperatures.

## Materials and methods

### Field cultivation test

We performed field cultivation test for 5 years (2013–2016 and 2018) at Nagano Vegetable and Ornamental Crops Experiment Station (Shiojiri city, Nagano prefecture, Japan; 36° 10′ N, 137° 93′ E). using a total of 10 cultivars of crisphead lettuce which consist of five cultivars of Empire type (cv ‘SummerAce’, cv ‘ShinanoPower’, cv ‘Patriot’, cv ‘Olympia’, and cv ‘ShinanoHope’) and five cultivars of Salinas type (cv ‘Lushina66’, cv ‘Mayer’, cv ‘Raptor’, cv ‘Vlettuce’, and cv ‘ShinanoGreen’). About 15–20 days after sowing to the tray, the seedlings were transplanted by hand into the mulch-covered field. It took about 35–45 days from transplant to harvest survey. Detailed meteorological conditions on field cultivation test are described in Table S6. The Crisphead lettuce cv ‘VI185’ and cv ‘ShinanoGreen’ were developed by Nagano Vegetable and Ornamental Crops Experiment Station.

### Phenotypic evaluation

Bolting was determined by measuring the inner stem length of six individual plants in the field cultivation test. In F_2_ plants, bolting was nondestructively evaluated by the flowering day to collect seeds from each plant. The flowering day was defined as the day when the second flower bloomed because the first flower at the top sometimes blooms too early for phenotypic differences to be observed. Tipburn susceptibility was compared by the number of plants showing symptoms on the outside and inside of the crisphead for 30 and 10 individuals, respectively. Statistical analyses were performed using R^21^. Significant differences in bolting and tipburn incidence between the Empire type and Salinas type were evaluated using a *t*-test and Fisher’s exact test, respectively. Leaf marginal serration was determined by observation of leaves in individual plants.

### Linkage analysis based on ddRAD-seq

Ninety-six individuals of the F_2_ progeny were obtained from a cross between ‘VI185’ and ‘ShinanoGreen’. This population was used to investigate both flowering day and serration of leaf margins. The selfed F_3_ population was used to estimate the genotypes of F_2_ individuals with regard to leaf marginal serration. All the plant materials were grown and artificially pollinated in the greenhouse.

### ddRAD-seq analysis

Genomic DNA was extracted from leaves using the Nucleo-Spin Plant II Extract Kit (Machery-Nagel, Duren, Germany). The RAD-seq library construction was performed followed a previously described method^22^. Briefly, genomic DNA from all the samples (‘VI185’, ‘ShinanoGreen’, and 96 F_2_ population samples) was digested with PacI (New England Biolabs, Beverly, MA, USA) and NlaIII (New England Biolabs). After ligation of the adapters to both ends of digested DNA fragments, they were amplified by indexed primers and pooled for Illumina sequencing. The ddRAD-seq libraries were sequenced using the HiSeq2500 platform (Illumina, San Diego, CA, USA). Paired-end sequencing reads (100 bp × 2) were analyzed for ddRAD-seq tag extraction and counting as follows. Raw sequence data (FASTQ) in the present ddRAD-seq were deposited in the Sequence Read Archive under accession number PRJNA523045.

### Genotyping and linkage map construction

The procedure for RAD tag extraction from sequence reads to genotyping is illustrated in Fig. 3. Briefly, adapter-trimmed sequence reads using the QuasR package of R^21^ were defined as RAD tags, and their frequency was counted using the table function of R. For identification of alleles, tags appearing uniquely in either parental line (multiple counts for either parent but none in the other parent) were selected. In addition, common tags between parental lines were extracted to investigate areas without polymorphism. The sequences of these ‘VI185’-specific tags, ‘ShinanoGreen’-specific tags, and parent-common tags were mapped to reference genome sequences of lettuce using the default ALN mode of the Burrows–Wheeler Aligner (BWA)^23^. A pair of each parent-specific tags mapped at the same position of the genome sequence was determined to be an allele (biallelic tag). Genotypes of biallelic tag loci were determined in 96 F_2_ individuals by the presence or absence of each allelic tag. Biallelic tags having either missing value with more than half of F_2_ individuals or a *P*-value of <0.001 based on the chi-squared test were eliminated from the genotype data. Because the genotype data from the NGS approach had the drawback of a large amount of missing data^24,25^ in most cases, we applied the ABHgenotypeR package of R^25^ for post-data correction and imputation. The parameter of maxHapLength in ABHgenotypeR was adjusted to five. The linkage map was graphically visualized with MapChart^26^. The genetic map construction of the genetic distances and location of each biallelic marker were calculated using the AntMap program^27^.

Scripts for these analyses are deposited in https://github.com/KousukeSEKI/RAD-seq_scripts as (1) Parent_script.R, (2) RAD_mapping_script_BWA_aln_script.txt, (3) Map_list_script.R, (4) Population_tagcount_script.R, (5) Genotype_script.R, (6) Convert_ABH_script.R, and (7) Post_data_correction_script.R.

### Linkage analysis by QTL detection with CIM

QTL detection with CIM was conducted using the Haley–Knott regression of the R/qtl package in R^28^. The genome-wide LOD threshold at the 1% significance level was individually determined using a 10,000-permutation test for each trait. The proportion of phenotypic variance was calculated from the value at the peak, as indicated by CIM. A detailed script is described in CIM_script.R (https://github.com/KousukeSEKI/RAD-seq_scripts).

### Resequencing analysis and de novo assembly

Genomic DNA was extracted from young leaves of the parents (‘VI185’ and ‘ShinanoGreen’) using NucleoSpin Plant II (Machery-Nagel, Duren, Germany). The genomic DNAs were sheared to ~300 bp with Covaris (Covaris Inc., USA). The DNA-seq libraries were prepared with 600 ng sheared DNA using the KAPA Hyper Prep Kit for Illumina (Roche Sequencing Solutions Inc, USA) with the PCR-free method in accordance with the manufacturer’s instructions. Library quality control was performed with an Agilent 2100 BioAnalyzer (Agilent Technologies, USA). The average library size was ~450 bp. Library quantification was performed with quantitative real-time PCR, and the concentration of each library was adjusted to 10 nM. The prepared libraries were sequenced using the Illumina HiSeq 2500 system (Illumina, USA). Conversion of base call data to FASTQ files and adapter trimming were performed using bcl2fastq2 (Illumina, USA). The FASTQ files were deposited in SRA and are accessible under Sequence Read Archive accession number DRA008299. The FASTQ files were imported to the CLC Genomics Workbench (QIAGEN, USA)^29^ for subsequent analysis. The trim sequences tool in the suite was used to filter out low-quality bases (<Q30), and only those reads that showed a quality score of ≥30 were retained. Filtered sequence reads were mapped onto the *L. sativa* v8.0 genome (https://phytozome.jgi.doe.gov/pz/portal.html#!info?alias=Org_Lsativa_er) using the Map Reads Reference tool, and local realignment was performed using the Local Realignment tool. De novo assembly to detect structural variant was performed using the CLC Genomics Workbench de novo assembly tool with default parameter settings.

### Designing PCR-based markers and their amplification

Polymorphisms near the locus at 251.386–253.367 Mbp in linkage group 5 (LG5), including insert, deletion, and SNP, were used to set markers. The name of the primer was set to (Linkage group) _ (genome version) _ (genome position). Primers for amplifying the locus were designed using the Primer3 website (http://bioinfo.ut.ee/primer3-0.4.0/), whereas KOD FX (TOYOBO, Japan) was used for amplification. PCR was performed using 0.5 μL DNA template, 0.4 μL of each primer (50 μM), 2 μL dNTP (2 mM), 5 μL 2× PCR Buffer, 0.2 μL KOD FX (1 U/μL), and distilled water (dH_2_O) to a final volume of 10 μL. PCR conditions were as follows: 94 °C for 5 min, 30 cycles of 94 °C for 30 s, and 58 °C for 30 s, followed by one cycle at 72 °C for 4 min. After amplification, 9 μL of PCR products was electrophoresed on 2% agarose gel (Takara-bio, Japan) at 100 V.

### Transcriptome analysis

Total RNA was extracted from individually collected triplicate leaves of ‘VI185’ and ‘ShinanoGreen’ using the NucleoSpin RNA Plant (Takara-bio, Japan). The RNA-seq libraries were prepared using the NEB Next Ultra RNA Library Prep Kit for Illumina (New England Biolabs, USA) in accordance with the manufacturer’s instructions. Library quality control was performed with an Agilent 2100 BioAnalyzer. The average insert size in the library was ~400 bp. Library quantification was performed with quantitative real-time PCR, and the concentration of each library was adjusted to 10 nM. The prepared libraries were sequenced using an Illumina HiSeq 2500 system. Conversion of base call data to FASTQ files and adapter trimming were performed using bcl2fastq2. The FASTQ files were deposited in DDBJ SRA and are accessible under Sequence Read Archive accession number DRA008298. The FASTQ files were imported to the CLC Genomics Workbench for subsequent analysis. The trim sequences tool in the suite was used to filter out low-quality bases (<Q30), and only those reads that showed a quality score of ≥30 were retained. Filtered sequence reads were mapped onto the *L. sativa* v8.0 genome using CLC Genomic Workbench with default parameters (Table S7). Expression values are reported as reads per kilobases per million values.

### Quantitative RT-PCR

Total RNA was extracted from leaves of 1-month-old plants of ‘ShinanoGreen’ and ‘VI185’ using RNeasy Plant Mini kit (Qiagen, USA). Three biologically replicated RNA samples were extracted from independent plants in each cultivar. First-strand cDNA was synthesized from each RNA sample using ProtoScript Reverse transcriptase (New England Biolabs, Ipswich MA) and random hexamer. For specific amplification of *LsTCP4* cDNA, a primer set (5′-ACGACGGCATCTCCGATAAG-3′ and 5′-ACCAGTGATGACTGAAGAACCT-3′) was designed. As the reference of quantitative PCR, primers for amplifying *TUB* gene encoding tubulin^30^ were employed. Each cDNA was amplified by Thermal Cycler Dice Real Time System (Takara-bio, Japan) using 2x TB Green Premix Ex Taq (Takara-bio, Japan) and Ct value was calculated using second derivative maximum (SDM) value. Based on Ct (SDM) values, relative expression level of *LsTCP4* to the reference gene (*TUB*) was calculated using the relative standard curve method in each sample.

### Electron microscopic observation of the leaf surface

For SEM (VE-7800; Keyence, Tokyo, Japan) observations, fresh leaves of the Empire and Salinas type were collected and mounted on a pedestal with carbon tape. The epidermal cells of leaf surface was visualized using SEM.

## Supplementary information

Supplementary Information

Supplementary Figure Legends

Supplementary Figure Legends

Supplementary Tables

Supplementary Tables

## Data Availability

Reference Sequence of *Lactuca sativa* L. used in this study can be found from Phytozome (DOE-JGI, http://phytozome.jgi.doe.gov/). The High throughput sequencing data are deposited to Sequence Read Archive with the accession number PRJNA523045 (ddRAD-seq), and to DDBJ Sequence Read Archive with the accession number DRA008299 (DNA-seq), DRA008298 (RNA-seq). The sequence of *LsTCP4* and the insertion sequence is available in Fig. S5. *LsTCP4* sequence data also can be found in the DDBJ/EMBL/GenBank under accession number LC480520.
