# Supplementary material for: A CIN-like TCP transcription factor (LsTCP4) having retrotransposon insertion associates with a shift from Salinas type to Empire type in crisphead lettuce (Lactuca sativa L.)
Source: Hortic Res. 2020 Feb 1;7:15. doi: 10.1038/s41438-020-0241-4 (PMC6994696; doi:10.1038/s41438-020-0241-4)
Supplement: Supplementary file 1 — Supplementary Information [file 41438_2020_241_MOESM1_ESM.docx]

Supplementary Information

A CIN-like TCP transcription factor (*LsTCP4*) having retrotransposon insertion associates with a shift from Salinas type to Empire type in crisphead lettuce (*Lactuca sativa* L.)

“Figure S1-S6.docx” file includes: Supplementary Fig. S1 to S6.

“Table S1-S7.xlsx” file includes: Supplementary Table S1 to S7.
