# Supplementary material for: A CIN-like TCP transcription factor (LsTCP4) having retrotransposon insertion associates with a shift from Salinas type to Empire type in crisphead lettuce (Lactuca sativa L.)
Source: Hortic Res. 2020 Feb 1;7:15. doi: 10.1038/s41438-020-0241-4 (PMC6994696; doi:10.1038/s41438-020-0241-4)
Supplement: Supplementary file 2 — Supplementary Figure Legends [file 41438_2020_241_MOESM2_ESM.docx]

**Supplementary Information**

**A CIN-like TCP transcription factor (*LsTCP4*) having retrotransposon insertion associates with a shift from Salinas type to Empire type in crisphead lettuce (*Lactuca sativa* L.)**

**This file includes:**

Supplementary Fig. S1 to S6


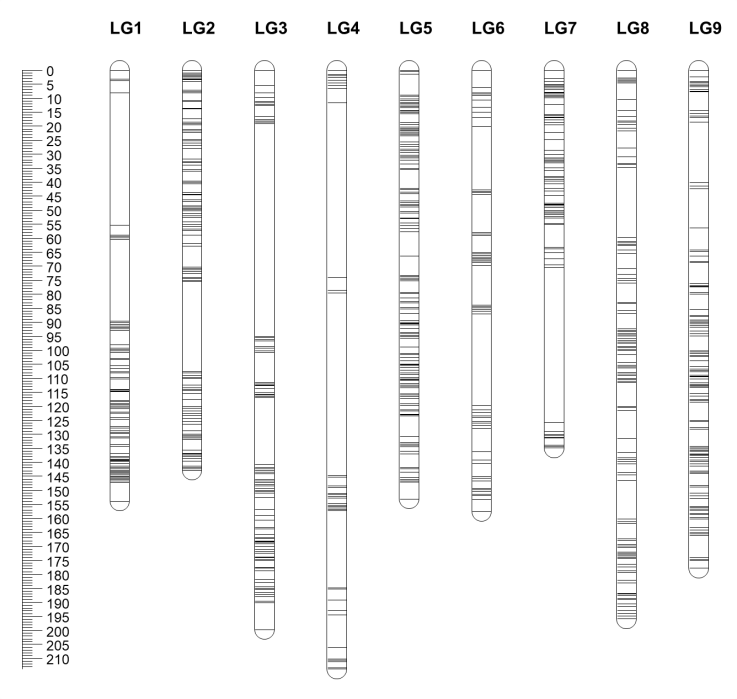


Fig. S1

Shematic representation of the consensus map for an F_2_ population derived from a cross between ‘VI185’ x ‘ShinanoGreen’. Ruler on left indicated the cM distance and the horizontal lines across the chromosomes indicated locus positions on each chromosome.


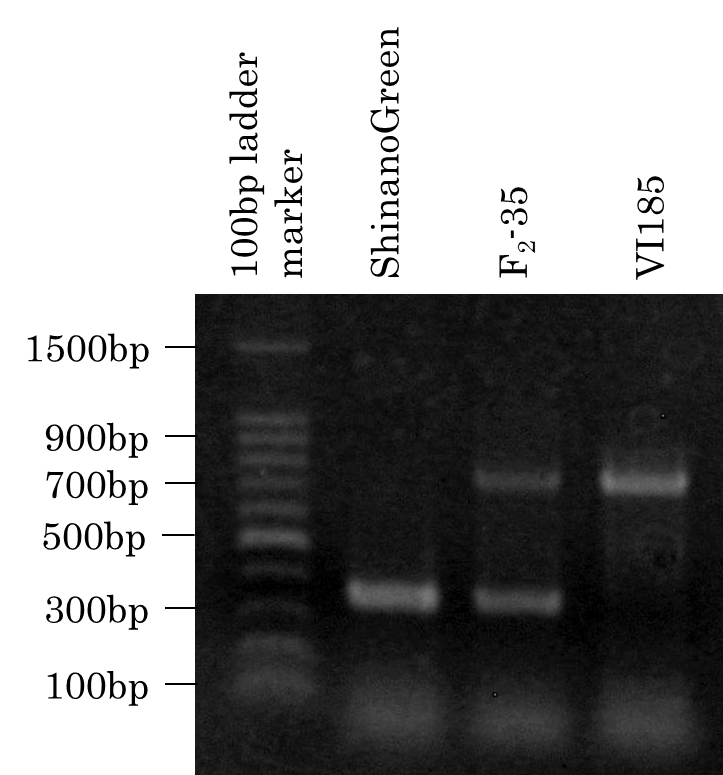


Fig. S2

Genotyping of ‘ShinanoGreen’ (Salinas type), ‘F_2_-35’ (Hetero), and ‘VI185’ (Empire type) using codominant Indel marker *LG5_v8_252.743Mbp*. In the F_3_ progeny of F_2_-35 showing wavy leaf phenotype, the leaf phenotype were segregated to wavy leaf and serrated leaf. PCR product was amplified with genomic DNA as template and separated on agarose gel.


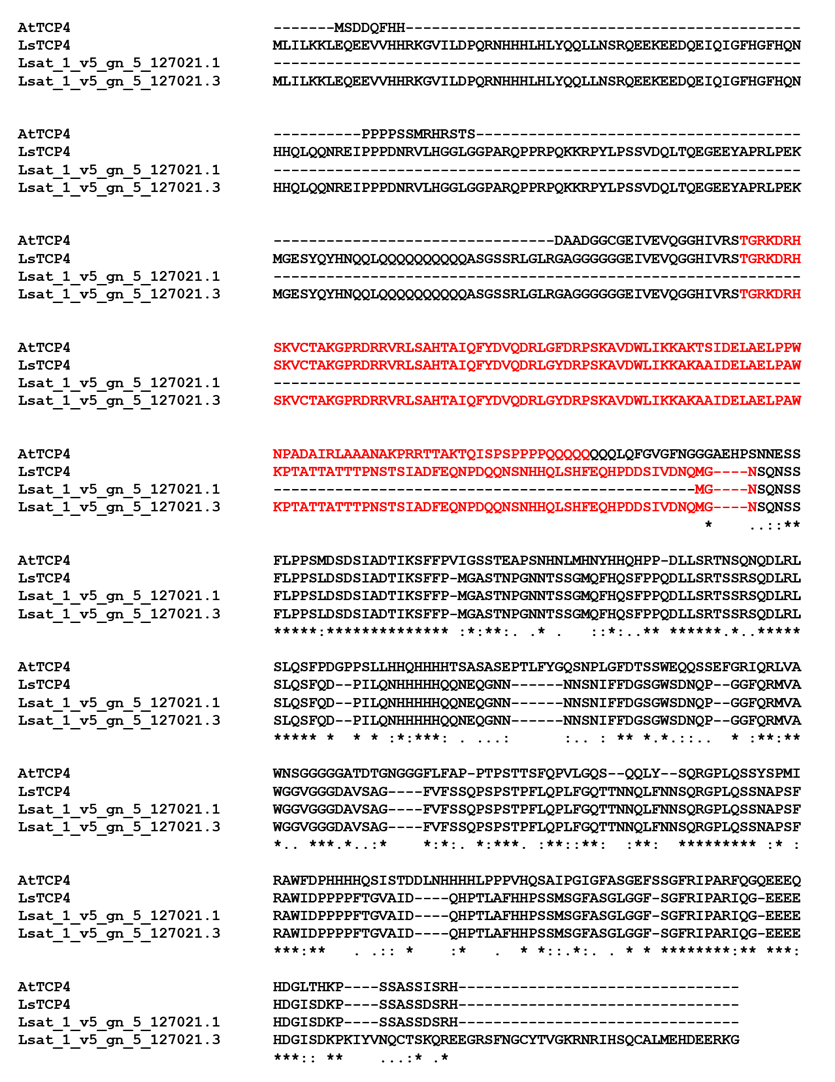


Fig. S3

Comparison of the amino acid sequences of *A.thaliana* TCP4 and *L. sativa* CIN-like TCP proteins. Amino acid sequences of *AtTCP4*, *LsTCP4*, *Lsat_1_v5_gn_5_127021.1* and *Lsat_1_v5_gn_5_127021.3* were aligned using the MUSCLE program (https://www.ebi.ac.uk/Tools/msa/muscle/). Identical amino acids are marked with asterisks (*), strongly similar amino acids are marked with two dots (:), and weakly similar amino acids are marked with one dot (·). Putative TCP domain were predicted using Pfam program (http://pfam.xfam.org). The amino acid sequences characters corresponding to putative TCP domain highlighted with red color.


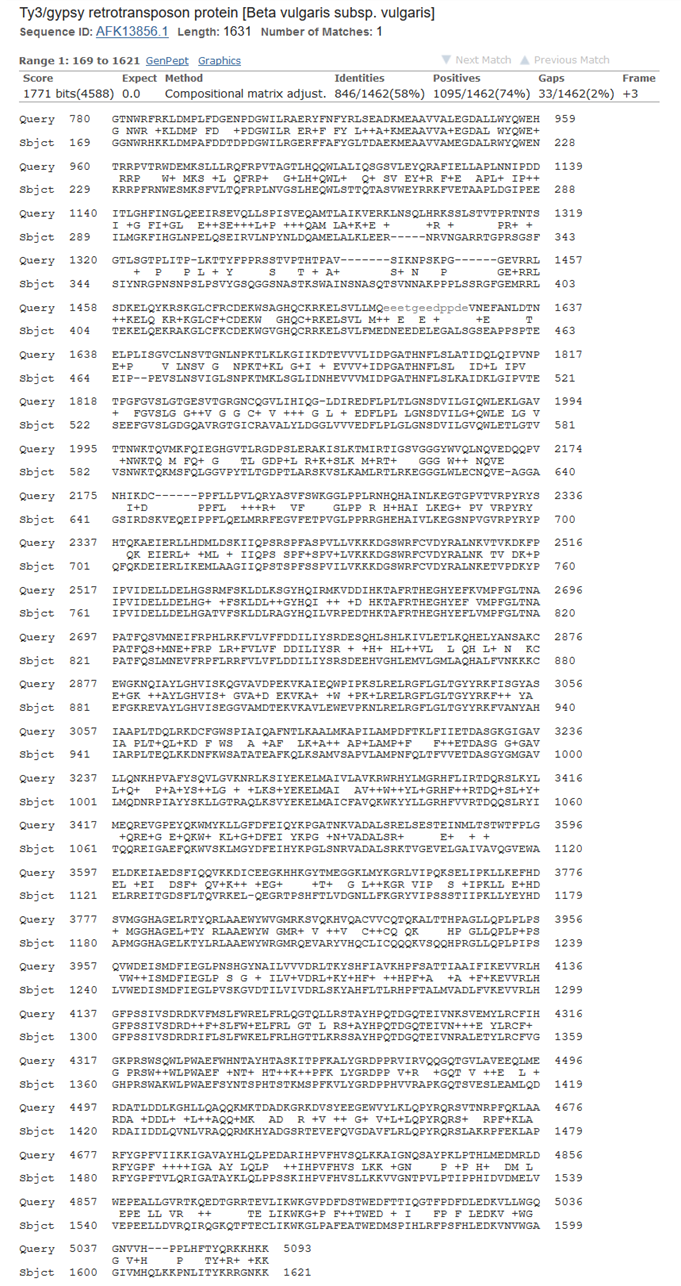


Fig. S4

Result of BLASTX against NCBI non-redundant protein database with query sequence as the insertion sequence in *Lsat_1_v5_gn_5_127021* of VI185.

ATGTTGATTTTGAAAAAGCTGGAACAGGAGGAGGTTGTTCATCATCGTAAGGGTGTGATTCTGGATCCTCAGAGGAATCATCATCATCTTCATCTGTATCAACAACTATTGAATTCAAGACAAGAAGAGAAGGAAGAAGATCAAGAAATTCAGATAGGTTTTCATGGATTCCATCAAAATCATCATCAGCTTCAACAAAATCGAGAAATCCCACCTCCCGACAACCGTGTTCTCCACGGAGGATTAGGTGGTCCGGCAAGGCAGCCGCCGCGGCCACAGAAGAAACGCCCTTATTTACCTTCTTCTGTGGACCAACTGACCCAAGAAGGAGAAGAATATGCACCGAGGCTGCCGGAAAAAATGGGAGAAAGTTATCAGTATCACAACCAACAACTCCAGCAGCAGCAGCAGCAGCAGCAACAGCAAGCAAGTGGGTCGTCGAGATTGGGATTGAGGGGCGCAGGTGGCGGAGGCGGTGGCGAGATTGTGGAAGTTCAGGGCGGTCACATTGTACGATCCACTGGCCGGAAAGACCGACACAGCAAGGTGTGCACAGCGAAAGGACCAAGGGACCGCCGTGTTCGTCTCTCGGCTCACACCGCCATCCAATTCTACGACGTCCAAGACCGCCTTGGTTACGACCGCCCAAGCAAAGCCGTCGATTGGCTTATTAAAAAAGCCAAGGCTGCCATTGATGAACTCGCGGAGCTTCCGGCATGGAAGCCCACTGCCACTACGGCGACGACAACCCCAAATTCAACATCGATTGCAGATTTTGAGCAAAACCCAGATCAACAAAACTCAAATCATCATCAACTAAGTCATTTCGAACAACACCCAGATGATAGTATTGTTGATAATCAAATGGGTAATTCACAAAACTCAAGCTTCTTGCCTCCGTCTCTTGATTCCGACTCTATAGCTGATACAATCAAGTCATTTTTTCCGATGGGTGCTTCAACAAATCCAGGAAATAATACTTCTTCAGGTATGCAATTTCATCAAAGTTTTCCACCTCAGGATTTGCTTTCAAGAACCAGTAGTCGAAGTCAAGATCTGAGGCTTTCTCTTCAATCATTCCAAGATCCGATTCTCCAGAACCACCACCATCACCACCAACAAAACGAACAGGGTAATAATAACAATAGCAATATATTCTTTGACGGGTCGGGTTGGTCCGACAACCAGCCTGGTGGGTTTCAAAGAATGGTGGCGTGGGGTGGTGTAGGAGGCGGAGACGCTGTTTCCGCCGGATTTGTCTTCAGTTCGCAGCCGTCGCCGTCGACGCCTTTTCTGCAACCGTTGTTCGGTCAAACAACAAACAATCAGTTATTCAACAATTCTCAGAGGGGACCCCTTCAGTCCAGTAACGCACCTTCGTTTCGTGCTTGGATCGACCCGCCTCCACCGTTCACCGGTGTCGCCATCGATCAACACCCAACCCTAGCTTTCCATCACCCATCTTCCATGTCCGGTTTCGCCTCCGGTTTAGGTGGGTTTTCCGGGTTTCGTATTCCAGCACGAATTCAAGGTGAAGAGGAGGAACACGACGGCATCTCCGATAAGCCGTCCTCTGCATCCTCCGATTCTCGCCATTGAGTAAAAGAAACCCATCACCTGTTTCTCAATTCCGCCTGATTTCGTTTTATTCTTAATTTCATCAATAATTACAGATCATCTTCATCCTCCATAATCCCTCTCAAACAGGAAAATTTACGTCAATCAATGTACTTCAAAGCAAAGAGAGGAGGGGAGAAGCTTCAATGGCTGCTACACAGTGGGAAAAAG**AAACCGAATCCATTCACAGG**TTCTTCAGTCATCACTGGTGTTTTTACGATTTAGCTTTCATGTTTGAATAGAGCTCTTTGTTTAGCTCTAAACCCTGTTTTAAATTATCTGGATTTAATGTTTTTAGACCATGTATGTGACTGTGTGTTATGAACTCAGATTCTCTCAAGGATAAATTAAAACCACAACTGATGGATTCACACTTCTTCTGGGATTAATGAACGAAAACAGAGATTACACAAGAAATTGATAAGAAAAAGAATGAAATAGAAATAGCAATTCGAGCTGCTACGGACTCCCAGAGAAGTAGAAACTTCTAATATTCTTCCATGCCATCACCAGGCTCCCTACCCATCCTTATAACGGAATGTCAGTATTTCTCTAAATTCCCACTTTGCCCCTGCTCCATGTCAGCAACATCCTCCTGGGTGATAGTGGTCCCTTGTCCTTTTAGAATCTTTTTGTGTTTCTTTCTTTGGTACGTGAAATGTAGTGGTGGGTGCACCACATTACCCTGCCCCCAAAGCAAGACCTTGTCCTCAAGGTCAAAGTCTGGAAAGGTGCCTTGAATAGTTGTGAAATCTTCCCAGGTGGAATCAAAATCAGGAACCCCCTTCCATTTGATCAACACTTCTGTTCTTCGACCTGTGTCTTCTTGCTTGGTACGAACCCCCAGCAATGCTTCTGGTTCCCAGTCAAGACGCATGTCTTCCATTAGGTGGGTTGGCAATTTCGGGTATGCTGACTGATTGCCTATTGCTTTCTTGAGTTGCGAAACATGAAAGACCGGATGGATCCTAGCATCCTCTGGTAGCTGTAAGTGATAAGCAACCGCACCAATTTTTTTGATAATCACGAATGGTCCATAAAATCGAGCCGCCAGCTTCTGGAAAGGTCTATTTGTCACTGACCTTTGTCTATACGGTTGCAATTTTAGATAGACCCACTCTCCTTCTTCATATGACACATCTTTTCTACCCTTATCAGCATCTGTCTTCATTTTTTGTTGAGCCTGAAGCAAGTGGCCCTTAAGATCATCCAACGTGGCGTCTCTTTCCATCAGTTGTTCTTCCACAGCCAAAACTCCTGTTTGCCCTTGCTGAACCCGTATAACACGCGGCGGGTCACGCCCATACAAGGCCTTAAAAGGCGTGATTTTGCTGGCGGTGTGATAAGCGGTGTTGTGCCAAAACTCTGCCCATGGTAACCATTGCGACCAGGAACGAGGCTTACCGTGGATGAAACACCTTAAATACATCTCCACCGACTTGTTGACGATCTCCGTTTGTCCATCGGTTTGCGGGTGGTAGGCTGTGCTTCTTAATAACTGAGTCCCTTGTAATCGAAACAATTCACGCCAAAACAAACTCATAAACACCTTGTCTCGATCAGAGACTATGGAAGATGGAAACCCATGAAGACGAACTACTTCCTTAATGAAAATTGCTGCAATTGTTGTTGCTGAGAAGGGGTGTTTAACCGCTATGAAATGGGAGTACTTAGTAAGGCGATCAACTACAACCAATATGGCATTGTAACCATGCGAGTTAGGTAGGCCTTCGATAAAATCCATGGATATCTCATCCCAAACCTGGCTCGGAAGGGGTAATGGTTGTAATAACCCCGCCGGATGAGTAGTTAAAGCTTTTTGGGTTTGGCACACGACACAGGCTTGTACATGTTTCTGGACACTCTTCCTCATTCCTACCCAATACCACTCCGCCGCCAACCTTTGGTAAGTTCTTAATTCCCCTGCATGTCCTCCCATCACCGAATCATGGAATTCCTTCAATAGCTTGGGAATCAACTCCGATTTTTGAGGTATGACCAACCTACCTTTATACATCAATTTCCCACCTTCCATTGTGTAGCCTTTGTGGTGTTTTCCTTCCTCACATATGTCTTTTTTTACCTGCTGAATGAAGGAATCTTCAGCTATTTCTTTATCCAATTCTCCCAACGGGAAGGTCCAAGTAGATGTAAGCATGTTGATTTCGGTTGATTCGGAGAGTTCTCGAGAGAGAGCATCTGCCACCTTATTGGTAGCCCCCGGTTTGTACTGAATCTCAAAGTCGAAGCCCAATAATTTATACATCCATTTTTGGTATTCTGGGCCCACTTCTCGTTGTTCCATCAAATATTTGAGACTGCGTTGGTCTGTTCTAATTAAAAAATGGCGTCCCATCAAGTAATGTCTCCACCTTTTAACAGCCAAAACAATGGCCATCAATTCCTTTTCATAAATCGATTTAAGCCTATTCTTTACCCCTAAAACTTGGCTATAAAAAGCCACTGGATGCTTGTTTTGCAGCAGGACTGCTCCTATCCCTTTTCCGGAAGCATCCGTTTCAATGATAAACAATTTGGTAAAGTCCGGCATGGCCAATATTGGTGCTTTCATTAGGGCTGCCTTTAGAGTGTTGAAAGCTTGGATGGCGATTGGTGACCACCCAAAGCAATCCTTGCGTAATTGATCCGTCAGAGGTGCTGCAATACTCGCATACCCTGAAATAAACTTTCTGTAATATCCTGTGAGACCCAGGAACCCCCTGAGTTCACGCAAAGATTTTGGAATGGGCCATTGTTCAATTGCTTTTACCTTTTCAGGGTCGACTGCTACCCCTTGTTTTGAAATAACATGGCCTAAGTAGGCTATTTGGTTTTTACCCCATTCACACTTGGCTGAATTAGCATATAACTCGTGTTGCTTCAATGTTTCCAAAACAATCTTCAAATGGGACAAATGTTGACTTTCGTCTCGGCTATAAATTAGGATGTCGTCAAAGAATACCAGCACAAACTTTCGTAGATGAGGTCTGAAAATCTCATTCATGACAGATTGAAAGGTGGCTGGGGCATTAGTAAGGCCGAAGGGCATAACCTTGAACTCATAATGCCCTTCATGCGTTCTAAACGCTGTTTTATGAATATCGTCCACCTTCATACGGATTTGATGATAACCTGATTTCAAATCCAGCTTGGAAAACATCCGTGATCCGTGTAATTCATCTAAAAGCTCGTCTATCACGGGTATGGGAAACTTGTCTTTAACCGTCACCTTGTTAAGGGCCCGATAGTCAACACAGAACCTCCACGAGCCATCTTTTTTTTTAACCAACAACACGGGGCTAGCAAAAGGACTTCGGGACGGTTGGATGATTTTGGAGTCGAGCATGTCGTGAAGCAATCTTTCAATTTCTGCTTTTTGGGTATGAGAGTACCTATAAGGACGTACCGTCACCGGTCCAGTTCCCTCCTTCAGATTAATGGCGTGCTGGTGATTCCTTAGTGGGGGTAGGCCTCCTTTCCAAGAAAAAACAGAAGCATACCTCTGTAATACTGGAAGTAGAAAGGGAGGGCAGTCTTTAATGTGATTCACGGGCTGTTGGTCCTCCACTTGGTTCAACTGGACCCAATATCCACCTCCCACAGAGCCAATGGTTCTAATCATGGTTTTTAAGGAAATTTTTGCCCTTTCTAAGGATGGGTCTCCCCGGAGAGTCACCCCGTGGCCTTCTATTTGAAACTTCATGACTTGCGTTTTCCAATTAGTAGTCACCGCCCCTAACTTCTCCAACCATTGGATTCCCAGAATTACGTCTGAATTTCCCAAGGTGAGCGGTAAGAAATCCTCTCTAATATCCAGCCCTTGAATATGTATTAATACCCCTTGGCAATTTCCTCTTCCGGTCACTGATTCTCCTGTGCCCAAAGAAACCCCAAACCCCGGAGTAGGGTTGACTGGAATCTGTAATTGATCTATAGTTGCCAAGGAAAGGAAATTGTGAGTTGCTCCCGGATCAATGAGTACCACCACTTCTGTGTCCTTAATGATTCCCTTAAGTTTGAGGGTCTTAGGATTGAGGTTACCAGTAACTGAATTCAAACAGACACCTGATATCAATGGGAGTTCGTTAGTGTCCAAATTTGCAAATTCATTGACTTCGTCTGGTGGGTCTTCTTCCCCTGTTTCTTCTTCTTGCATTAAGAGTACACTGAGTTCCTTCCTCTTGCATTGATGCCCTGCACTCCATTTCTCATCACAACGAAAGCAAAGCCCTTTTGATCGTTTATATTGCAGTTCTTTATCCGACAATCTTCTCACTTCCCCGCCTGGTTTGCTGGGGTTCTTAATCGACACGGCTGGGGTGTGGGTTGGTACCGTAGACGATCGAGGGGGGAAATAAGTGGTTTTGAGCGGGGTTATCAGGGGTGTTCCAGAAAGAGTACCTGATGTGTTAGTCCGAGGAGTTACAGTGGAGAGAGAGGATTTCCGATGGAGTTGGGAATTAAGCTTGCGTTCGACTTTGATGGCCAACGTCATAGCTTGTTCCACTGATATGGGGCTCAATAACTGGACCTCAGACCTAATTTCCTCCTGTAGCCCGTTGATGAAGTGGCCCAAGGTGATGTCATCAGGTATGTTGTTTAATGGGGCAAGTAGTTCAATAAAGGCTCGTTGGTATTCTAAAACCGACCCAGATTGAATTAGGGCTAACCACTGCTGGTGTAACGTACCTGCAGTAACCGGACGAAACTGTCGTAATAGGAGAGATTTCATCTCATCCCACCGTGTCACCGGCCGGCGAGTGTGCTCCCACTGGTACCAGAGAAGGGCGTCTCCTTCAAGAGCCACCACCGCTGCCTCCATTTTATCAGCTTCTGATAATCTGTAGAAATTGAAATAGCGTTCTGCACGTAATATCCATCCATCGGGGTTTTCCCCGTCGAAGAGGGGCATATCGAGTTTCCGAAATCGCCAATTGGTCCCTCCTGTTGGTTCACCGTTATTGGTTCGGCCGTTGCCCCCATCGCCACTACCTCCCGACCCACCTACGACGCCGCTGCGGTATCCCCCAGCGGATTCTTTTGGTGTCGCCAAAATGGAAGAAACAGGTTCGGACTCCTTCTCCCCTTCTCCTGACTTCATCCCTTTAGTTACAGCTCTCAAAACCTCTTCCAACTTCTGGTCCATCTTAAGCTGATTTTGGTCCATCTTCAGCTGGATGGAAG**CTATGGAGTCCGCCTGATGT**TGGATCTGATTACCTTGGGTCTCTAAGCGATCATACAACACACCTAATTCTGACTCCAGCGATTCAACCCTCTTGAGTTGAGCTCCTCCAGCCTCTTTCCCACCTCCCTTTTCTCCAGCCATAATCCCAGGTGGTTGGTTGCTCTGATACCAATTTGTTATGAACTCAGATTCTCTCAAGATAAATTAAAACCACAACTGATGGATTCACACTTCTTCTGGGATTAATGAACGAAAACAGAGATTACACAAGAAATTGATAAGAAAAAGAATGAAATAGAAATAGCAATTCGAGCTGCTACGGACTCCCAGAGAAGTAGAAACTTCTAATATTCTTCCATGCCATCACCAGGCTCCCTACCCATCCTTATAACGGAATGTCAGTATTTCTCTAAATTCCCACTTTGCCCCTGCTCCATGTCAGCAACATCCTCCTGGGTGATAGTGGTCCCTTGTCCTTTTAGAATCTTTTTGTGTTTCTTTCTTTGGTACGTGAAATGTAGTGGTGGGTGCACCACACTGTGCCAATTGCCTTCTTTTGCTATATAATTATCAAAAATACTCTGCTAAAAACAGAGCAAGTGATTTGTTTTGGTTATAATTTCACTGTTATTCGCCCAACTTTAGTAAAACAAGAATCTTTTGAATATCAACCAAATCAACACATTCTTTGTGTGTGATTGATTGAAATTTGAAAGGCCATGA**GATGAGAGGAGCAAATGGGA**TGTTTCCCTTTGTTTAGAAAATTGTCAATTTTTTATGTATGTGGGGTTTGAAAGAGAGAGCTCCAATTTTTGTCTCTCTTTTTACCCAATTCTTGCAATCTTGCTTCATATGCTTTTTCATTAGTGTGCCCTAATGGAACATGATGAAGAAAGGAAAGGGTAG

_____ *LsTCP4* CDS

_____ *LsTCP4* 3’UTR

_____ *LsTCP4* intron

_____ Ty3/gypsy retrotransposon-like insertion

_____ 3’flanking sequence of *LsTCP4*

Fig. S5.

Sequence of *LsTCP4*, Ty3/gypsy retrotransposon like insert and position of LG5_v8_252.743Mbp primers.


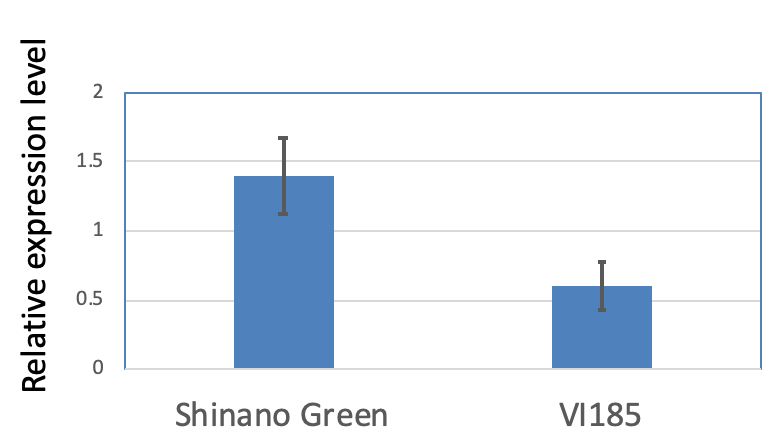


Fig. S6

Comparison of *LsTCP4* expression in the leaves between ShinanoGreen and VI185 by quantitative RT-PCR. Quantitative RT-PCR analysis of *LsTCP4* gene was carried out in each three replicated plants of ShinanoGreen and VI185. *LsTCP4* expression level was normalized by the expression of *TUB* gene in each sample. Normalized expression value in one replicate of ShinanoGreen was defined as the standard (=1) and relative expression level in other samples were calculated. Averaged relative expression level and standard error among triplicates were indicated.
