# Supplementary material for: A CIN-like TCP transcription factor (LsTCP4) having retrotransposon insertion associates with a shift from Salinas type to Empire type in crisphead lettuce (Lactuca sativa L.)
Source: Hortic Res. 2020 Feb 1;7:15. doi: 10.1038/s41438-020-0241-4 (PMC6994696; doi:10.1038/s41438-020-0241-4)
Supplement: Supplementary file 3 — Supplementary Figure Legends [file 41438_2020_241_MOESM3_ESM.pdf]

Supplementary Information

A CIN-like TCP transcription factor (*LsTCP4*) having retrotransposon insertion associates with a shift from Salinas type to Empire type in crisphead lettuce (*Lactuca sativa* L.)

This file includes:

Supplementary Fig. S1 to S6

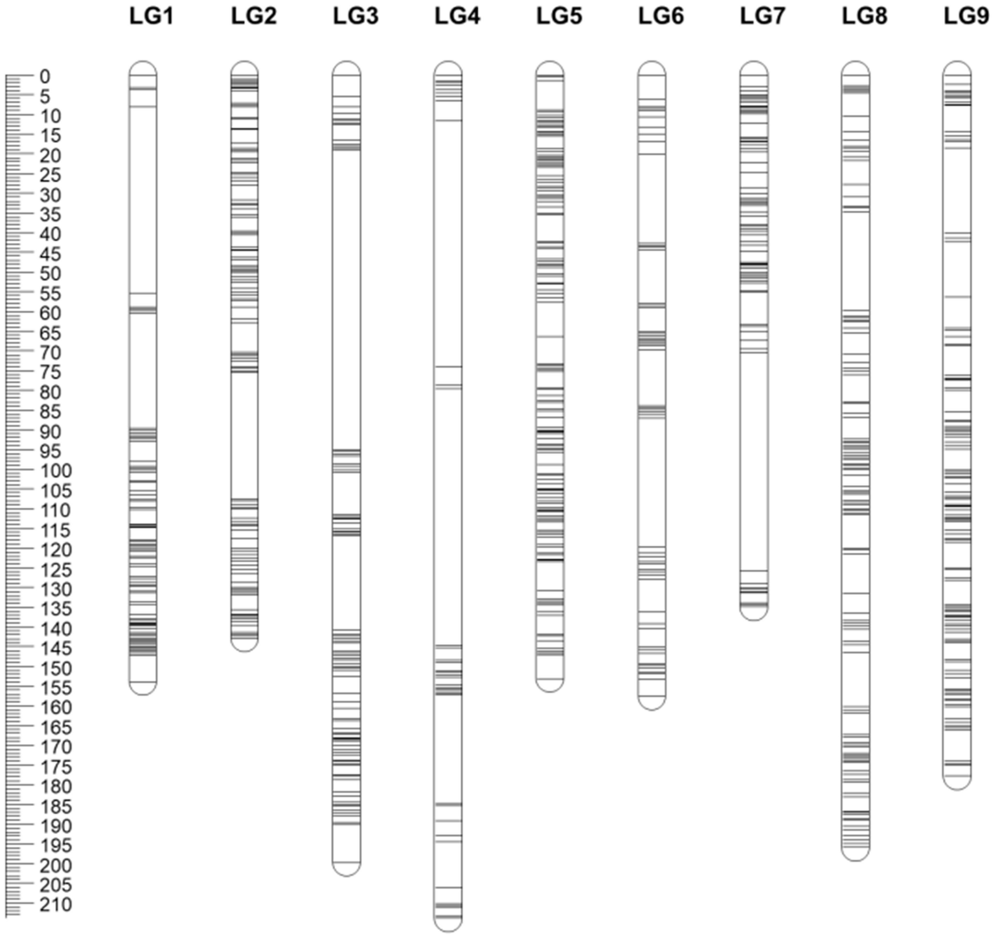

Fig. S1

Shematic representation of the consensus map for an F_2 population derived from a cross between ‘VI185’ x ‘ShinanoGreen’. Ruler on left indicated the cM distance and the horizontal lines across the chromosomes indicated locus positions on each chromosome.

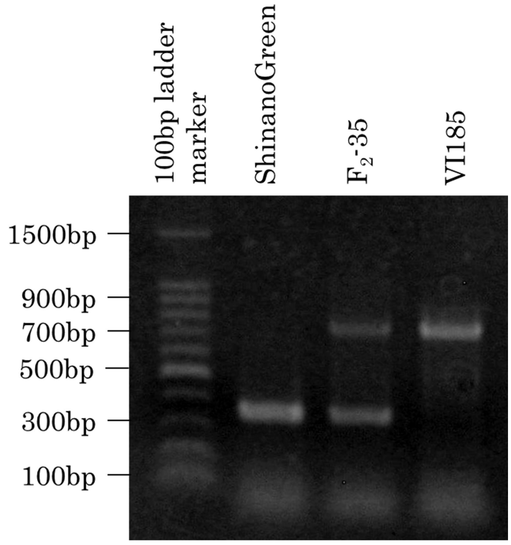

Fig. S2

Genotyping of ‘ShinanoGreen’ (Salinas type), ‘F_2-35’ (Hetero), and ‘VI185’ (Empire type) using codominant Indel marker *LG5_v8_252.743Mbp*. In the F_3 progeny of F_2-35 showing wavy leaf phenotype, the leaf phenotype were segregated to wavy leaf and serrated leaf. PCR product was amplified with genomic DNA as template and separated on agarose gel.

```
AtTCP4                    -------MSDDQFHH---------------------------------------------
LsTCP4                    MLILKKLEQEEVVHHRKGVILDPQRNHHHLHLYQQLLNSRQEEKEEDQEIQIGFHGFHQN
Lsat_1_v5_gn_5_127021.1   ------------------------------------------------------------
Lsat_1_v5_gn_5_127021.3   MLILKKLEQEEVVHHRKGVILDPQRNHHHLHLYQQLLNSRQEEKEEDQEIQIGFHGFHQN

AtTCP4                    ----------PPPPSSMRHRSTS-------------------------------------
LsTCP4                    HHQLQQNREIPPPDNRVLHGGLGGPARQPPRPQKKRPYLPSSVDQLTQEGEEYAPRLPEK
Lsat_1_v5_gn_5_127021.1   ------------------------------------------------------------
Lsat_1_v5_gn_5_127021.3   HHQLQQNREIPPPDNRVLHGGLGGPARQPPRPQKKRPYLPSSVDQLTQEGEEYAPRLPEK

AtTCP4                    --------------------------------DAADGGCGEIVEVQGGHIVRSTGRKDRH
LsTCP4                    MGESYQYHNQQLQQQQQQQQQQASGSSRLGLRGAGGGGGGEIVEVQGGHIVRSTGRKDRH
Lsat_1_v5_gn_5_127021.1   ------------------------------------------------------------
Lsat_1_v5_gn_5_127021.3   MGESYQYHNQQLQQQQQQQQQQASGSSRLGLRGAGGGGGGEIVEVQGGHIVRSTGRKDRH

AtTCP4                    SKVCTAKGPRDRRVRLSAHTAIQFYDVQDRLGFDRPSKAVDWLIKKAKTSIDELAELPPW
LsTCP4                    SKVCTAKGPRDRRVRLSAHTAIQFYDVQDRLGYDRPSKAVDWLIKKAKAAIDELAELPAW
Lsat_1_v5_gn_5_127021.1   ------------------------------------------------------------
Lsat_1_v5_gn_5_127021.3   SKVCTAKGPRDRRVRLSAHTAIQFYDVQDRLGYDRPSKAVDWLIKKAKAAIDELAELPAW

AtTCP4                    NPADAIRLAAANAKPRRTTAKTQISPSPPPPQQQQQQQQLQFGVGFNGGGAEHPSNNESS
LsTCP4                    KPTATTATTTPNSTSIADFEQNPDQQNSNHHQLSHFEQHPDDSIVDNQMG----NSQNSS
Lsat_1_v5_gn_5_127021.1   ------------------------------------------------MG----NSQNSS
Lsat_1_v5_gn_5_127021.3   KPTATTATTTPNSTSIADFEQNPDQQNSNHHQLSHFEQHPDDSIVDNQMG----NSQNSS
                                                                           *    ..::**

AtTCP4                    FLPPSMDSDSIADTIKSFFPVIGSSTEAPSNHNLMHNYHHQHPP-DLLSRTNSQNQDLRL
LsTCP4                    FLPPSLDSDSIADTIKSFFP-MGASTNPGNNTSSGMQFHQSFPPQDLLSRTSSRSQDLRL
Lsat_1_v5_gn_5_127021.1   FLPPSLDSDSIADTIKSFFP-MGASTNPGNNTSSGMQFHQSFPPQDLLSRTSSRSQDLRL
Lsat_1_v5_gn_5_127021.3   FLPPSLDSDSIADTIKSFFP-MGASTNPGNNTSSGMQFHQSFPPQDLLSRTSSRSQDLRL
                          *****:************** :*:**:. .* .   ::*:..** ******.*..*****

AtTCP4                    SLQSFPDGPPSLLHHQHHHHTSASASEPTLFYGQSNPLGFDTSSWEQQSSEFGRIQRLVA
LsTCP4                    SLQSFQD--PILQNHHHHHQQNEQGNN------NNSNIFFDGSGWSDNQP--GGFQRMVA
Lsat_1_v5_gn_5_127021.1   SLQSFQD--PILQNHHHHHQQNEQGNN------NNSNIFFDGSGWSDNQP--GGFQRMVA
Lsat_1_v5_gn_5_127021.3   SLQSFQD--PILQNHHHHHQQNEQGNN------NNSNIFFDGSGWSDNQP--GGFQRMVA
                          ***** *  * * :*:***: . ...:      :.. : ** *.*.::..  * :**:**

AtTCP4                    WNSGGGGGATDTGNGGGFLFAP-PTPSTTSFQPVLGQS--QQLY--SQRGPLQSSYSPMI
LsTCP4                    WGGVGGGDAVSAG----FVFSSQPSPSTPFLQPLFGQTTNNQLFNNSQRGPLQSSNAPSF
Lsat_1_v5_gn_5_127021.1   WGGVGGGDAVSAG----FVFSSQPSPSTPFLQPLFGQTTNNQLFNNSQRGPLQSSNAPSF
Lsat_1_v5_gn_5_127021.3   WGGVGGGDAVSAG----FVFSSQPSPSTPFLQPLFGQTTNNQLFNNSQRGPLQSSNAPSF
                          *.. ***.*..:*    *:*:. *:***. :**::**:  :**:  ********* :* :

AtTCP4                    RAWFDPHHHHQSISTDDLNHHHHLPPPVHQSAIPGIGFASGEFSSGFRIPARFQGQEEEQ
LsTCP4                    RAWIDPPPPFTGVAID----QHPTLAFHHPSSMSGFASGLGGF-SGFRIPARIQG-EEEE
Lsat_1_v5_gn_5_127021.1   RAWIDPPPPFTGVAID----QHPTLAFHHPSSMSGFASGLGGF-SGFRIPARIQG-EEEE
Lsat_1_v5_gn_5_127021.3   RAWIDPPPPFTGVAID----QHPTLAFHHPSSMSGFASGLGGF-SGFRIPARIQG-EEEE
                          ***:**   . .:: *    :*   .  * *::.*:. . * * ********:** ***:

AtTCP4                    HDGLTHKP----SSASSISRH--------------------------------
LsTCP4                    HDGISDKP----SSASSDSRH--------------------------------
Lsat_1_v5_gn_5_127021.1   HDGISDKP----SSASSDSRH--------------------------------
Lsat_1_v5_gn_5_127021.3   HDGISDKPKIYVNQCTSKQREEGRSFNGCYTVGKRNRIHSQCALMEHDEERKG
                          ***:: **    ...:* .*
```

Fig. S3

Comparison of the amino acid sequences of *A.thaliana* TCP4 and *L. sativa* CIN-like TCP proteins. Amino acid sequences of *AtTCP4*, *LsTCP4*, *Lsat_1_v5_gn_5_127021.1* and *Lsat_1_v5_gn_5_127021.3* were aligned using the MUSCLE program (https://www.ebi.ac.uk/Tools/msa/muscle/). Identical amino acids are marked with asterisks (*), strongly similar amino acids are marked with two dots (:), and weakly similar amino acids are marked with one dot (·). Putative TCP domain were predicted using Pfam program (http://pfam.xfam.org). The amino acid sequences characters corresponding to putative TCP domain highlighted with red color.

Ty3/gypsy retrotransposon protein [Beta vulgaris subsp. vulgaris]

Sequence ID: AFK13856.1 **Length:** 1631 **Number of Matches:** 1

Range 1: 169 to 1621 GenPept Graphics ▼ Next Match ▲ Previous Match

Score	Expect	Method	Identities	Positives	Gaps	Frame
1771 bits(4588)	0.0	Compositional matrix adjust.	846/1462(58%)	1095/1462(74%)	33/1462(2%)	+3

```
Query  780   GTNWRFRKLDMPLFDGENPDGWILRAERYFNFYRLSEADKMEAAVVALEGDALLWYQWEH  959
             G NWR +KLDMP FD  +PDGWILR ER+F FY L++A+KMEAAVVA+EGDAL WYQWE+
Sbjct  169   GGNWRHKKLDMPAFDDTDPDGWILRGERFFAFYGLTDAEKMEAAVVAMEGDALRWYQWEN  228

Query  960   TRRPVTRWDEMKSLLLRQFRPVTAGTLHQQWLALIQSGSVLEYQRAFIELLAPLNNIPDD  1139
              RRP   W+ MKS +L QFRP+  G+LH+QWL+  Q+ SV EY+R F+E  APL+ IP++
Sbjct  229   KRRPFRNWESMKSFVLTQFRPLNVGSLHEQWLSTTQTASVWEYRRKFVETAAPLDGIPEE  288

Query  1140  ITLGHFINGLQEEIRSEVQLLSPISVEQAMTLAIKVERKLNSQLHRKSSLSTVTPRTNTS  1319
             I +G FI+GL  E++SE+++L+P +++QAM LA+K+E +     +R +      PR+ +
Sbjct  289   ILMGKFIHGLNPELQSEIRVLNPYNLDQAMELALKLEER-----NRVNGARRTGPRSGSF  343

Query  1320  GTLSGTPLITP-LKTTYFPPRSSTVPTHTPAV-------SIKNPSKPG------GEVRRL  1457
                +  P   P L + Y     S   T + A+       S+ N   P       GE+RRL
Sbjct  344   SIYNRGPNSNPSLPSVYGSQGGSNASTKSWAINSNASQTSVNNAKPPPLSSRGFGEMRRL  403

Query  1458  SDKELQYKRSKGLCFRCDEKWSAGHQCKRKELSVLLMQeeetgeedppdeVNEFANLDTN  1637
             ++KELQ KR+KGLCF+CDEKW  GHQC+RKELSVL M++ E  E +     +E     T
Sbjct  404   TEKELQEKRAKGLCFKCDEKWGVGHQCRRKELSVLFMEDNEEDELEGALSGSEAPPSPTE  463

Query  1638  ELPLISGVCLNSVTGNLNPKTLKLKGIIKDTEVVVLIDPGATHNFLSLATIDQLQIPVNP  1817
             E+P    V LNSV G  NPKT+KL G+I + EVVV+IDPGATHNFLSL  ID+L IPV
Sbjct  464   EIP--PEVSLNSVIGLSNPKTMKLSGLIDNHEVVVMIDPGATHNFLSLKAIDKLGIPVTE  521

Query  1818  TPGFGVSLGTGESVTGRGNCQGVLIHIQG-LDIREDFLPLTLGNSDVILGIQWLEKLGAV  1994
             +  FGVSLG G++V G G C+ V +++ G L + EDFLPL LGNSDVILG+QWLE LG V
Sbjct  522   SEEFGVSLGDGQAVRGTGICRAVALYLDGGLVVVEDFLPLGLGNSDVILGVQWLETLGTV  581

Query  1995  TTNWKTQVMKFQIEGHGVTLRGDPSLERAKISLKTMIRTIGSVGGGYWVQLNQVEDQQPV  2174
              +NWKTQ M FQ+ G   TL GDP+L R+K+SLK M+RT+   GGG W++ NQVE
Sbjct  582   VSNWKTQKMSFQLGGVPYTLTGDPTLARSKVSLKAMLRTLRKEGGGLWLECNQVE-AGGA  640

Query  2175  NHIKDC------PPFLLPVLQRYASVFSWKGGLPPLRNHQHAINLKEGTGPVTVRPYRYS  2336
               I+D       PPFL  +++R+  VF    GLPP R H+HAI LKEG+ PV VRPYRY
Sbjct  641   GSIRDSKVEQEIPPFLQELMRRFEGVFETPVGLPPRRGHEHAIVLKEGSNPVGVRPYRYP  700

Query  2337  HTQKAEIERLLHDMLDSKIIQPSRSPFASPVLLVKKKDGSWRFCVDYRALNKVTVKDKFP  2516
               QK EIERL+ +ML + IIQPS SPF+SPV+LVKKKDGSWRFCVDYRALNK TV DK+P
Sbjct  701   QFQKDEIERLIKEMLAAGIIQPSTSPFSSPVILVKKKDGSWRFCVDYRALNKETVPDKYP  760

Query  2517  IPVIDELLDELHGSRMFSKLDLKSGYHQIRMKVDDIHKTAFRTHEGHYEFKVMPFGLTNA  2696
             IPVIDELLDELHG+ +FSKLDL++GYHQI ++ +D HKTAFRTHEGHYEF VMPFGLTNA
Sbjct  761   IPVIDELLDELHGATVFSKLDLRAGYHQILVRPEDTHKTAFRTHEGHYEFLVMPFGLTNA  820

Query  2697  PATFQSVMNEIFRPHLRKFVLVFFDDILIYSRDESQHLSHLKIVLETLKQHELYANSAKC  2876
             PATFQS+MNE+FRP LR+FVLVF DDILIYSR + +H+ HL++VL  L QH L+ N  KC
Sbjct  821   PATFQSLMNEVFRPFLRRFVLVFLDDILIYSRSDEEHVGHLEMVLGMLAQHALFVNKKKC  880

Query  2877  EWGKNQIAYLGHVISKQGVAVDPEKVKAIEQWPIPKSLRELRGFLGLTGYYRKFISGYAS  3056
             E+GK ++AYLGHVIS+ GVA+D EKVKA+ +W +PK+LRELRGFLGLTGYYRKF++ YA
Sbjct  881   EFGKREVAYLGHVISEGGVAMDTEKVKAVLEWEVPKNLRELRGFLGLTGYYRKFVANYAH  940

Query  3057  IAAPLTDQLRKDCFGWSPIAIQAFNTLKAALMKAPILAMPDFTKLFIIETDASGKGIGAV  3236
             IA PLT+QL+KD F WS  A +AF  LK+A++ AP+LAMP+F   F++ETDASG G+GAV
Sbjct  941   IARPLTEQLKKDNFKWSATATEAFKQLKSAMVSAPVLAMPNFQLTFVVETDASGYGMGAV  1000

Query  3237  LLQNKHPVAFYSQVLGVKNRLKSIYEKELMAIVLAVKRWRHYLMGRHFLIRTDQRSLKYL  3416
             L+Q+  P+A+YS++LG + +LKS+YEKELMAI  AV++W++YL+GRHF++RTDQ+SL+Y+
Sbjct  1001  LMQDNRPIAYYSKLLGTRAQLKSVYEKELMAICFAVQKWKYYLLGRHFVVRTDQQSLRYI  1060

Query  3417  MEQREVGPEYQKWMYKLLGFDFEIQYKPGATNKVADALSRELSESTEINMLTSTWTFPLG  3596
              +QRE+G E+QKW+ KL+G+DFEI YKPG +N+VADALSR+     E+  + +
Sbjct  1061  TQQREIGAEFQKWVSKLMGYDFEIHYKPGLSNRVADALSRKTVGEVELGAIVAVQGVEWA  1120

Query  3597  ELDKEIAEDSFIQQVKKDICEEGKHHKGYTMEGGKLMYKGRLVIPQKSELIPKLLKEFHD  3776
             EL +EI  DSF+ QV+K++ +EG+    +T+  G L++KGR VIP  S +IPKLL E+HD
Sbjct  1121  ELRREITGDSFLTQVRKEL-QEGRTPSHFTLVDGNLLFKGRYVIPSSSTIIPKLLYEYHD  1179

Query  3777  SVMGGHAGELRTYQRLAAEWYWVGMRKSVQKHVQACVVCQTQKALTTHPAGLLQPLPLPS  3956
             + MGGHAGEL+TY RLAAEWYW GMR+ V ++V  C++CQ QK    HP GLLQPLP+PS
Sbjct  1180  APMGGHAGELKTYLRLAAEWYWRGMRQEVARYVHQCLICQQQKVSQQHPRGLLQPLPIPS  1239

Query  3957  QVWDEISMDFIEGLPNSHGYNAILVVVDRLTKYSHFIAVKHPFSATTIAAIFIKEVVRLH  4136
              VW++ISMDFIEGLP S G + ILV+VDRL+KY+HF+ ++HPF+A  +A +F+KEVVRLH
Sbjct  1240  LVWEDISMDFIEGLPVSKGVDTILVIVDRLSKYAHFLTLRHPFTALMVADLFVKEVVRLH  1299

Query  4137  GFPSSIVSDRDKVFMSLFWRELFRLQGTQLLRSTAYHPQTDGQTEIVNKSVEMYLRCFIH  4316
             GFPSSIVSDRD++F+SLFW+ELFRL GT L RS+AYHPQTDGQTEIVN+++E YLRCF+
Sbjct  1300  GFPSSIVSDRDRIFLSLFWKELFRLHGTTLKRSSAYHPQTDGQTEIVNRALETYLRCFVG  1359

Query  4317  GKPRSWSQWLPWAEFWHNTAYHTASKITPFKALYGRDPPRVIRVQQGQTGVLAVEEQLME  4496
             G PRSW++WLPWAEF +NT+ HT++K++PFK LYGRDPP V+R  +GQT V ++E  L +
Sbjct  1360  GHPRSWAKWLPWAEFSYNTSPHTSTKMSPFKVLYGRDPPHVVRAPKGQTSVESLEAMLQD  1419

Query  4497  RDATLDDLKGHLLQAQQKMKTDADKGRKDVSYEEGEWVYLKLQPYRQRSVTNRPFQKLAA  4676
             RDA +DDL+ +L++AQQ+MK  AD  R +V ++ G+ V+L+LQPYRQRS+  RPF+KLA
Sbjct  1420  RDAIIDDLQVNLVRAQQRMKHYADGSRTEVEFQVGDAVFLRLQPYRQRSLAKRPFEKLAP  1479

Query  4677  RFYGPFVIIKKIGAVAYHLQLPEDARIHPVFHVSQLKKAIGNQSAYPKLPTHLMEDMRLD  4856
             RFYGPF ++++IGA AY LQLP  ++IHPVFHVS LKK +GN    P +P H+  DM L
Sbjct  1480  RFYGPFTVLQRIGATAYKLQLPPSSKIHPVFHVSLLKKVVGNTPVLPTIPPHIDVDMELV  1539

Query  4857  WEPEALLGVRTKQEDTGRRTEVLIKWKGVPDFDSTWEDFTTIQGTFPDFDLEDKVLLWGQ  5036
              EPE LL VR  ++     TE LIKWKG+P F++TWED + I   FP F LEDKV +WG
Sbjct  1540  VEPEELLDVRQIRQGKQTFTECLIKWKGLPAFEATWEDMSPIHLRFPSFHLEDKVNVWGA  1599

Query  5037  GNVVH---PPLHFTYQRKKHKK  5093
             G V+H    P   TY+R+ +KK
Sbjct  1600  GIVMHQLKKPNLITYKRRGNKK  1621
```

Fig. S4

Result of BLASTX against NCBI non-redundant protein database with query sequence as the insertion sequence in *Lsat_1_v5_gn_5_127021* of VI185.

ATGTTGATTTTGAAAAAGCTGGAACAGGAGGAGGTTGTTCATCATCGTAAGGGTGTGATTCTG
GATCCTCAGAGGAATCATCATCATCTTCATCTGTATCAACAACTATTGAATTCAAGACAAGAAG
AGAAGGAAGAAGATCAAGAAATTCAGATAGGTTTTCATGGATTCCATCAAAATCATCATCAGC
TTCAACAAAATCGAGAAATCCCACCTCCCGACAACCGTGTTCTCCACGGAGGATTAGGTGGT
CCGGCAAGGCAGCCGCCGCGGCCACAGAAGAAACGCCCTTATTTACCTTCTTCTGTGGACCA
ACTGACCCAAGAAGGAGAAGAATATGCACCGAGGCTGCCGGAAAAAATGGGAGAAAGTTAT
CAGTATCACAACCAACAACTCCAGCAGCAGCAGCAGCAGCAGCAACAGCAAGCAAGTGGGT
CGTCGAGATTGGGATTGAGGGGCGCAGGTGGCGGAGGCGGTGGCGAGATTGTGGAAGTTCA
GGGCGGTCACATTGTACGATCCACTGGCCGGAAAGACCGACACAGCAAGGTGTGCACAGCG
AAAGGACCAAGGGACCGCCGTGTTCGTCTCTCGGCTCACACCGCCATCCAATTCTACGACGT
CCAAGACCGCCTTGGTTACGACCGCCCAAGCAAAGCCGTCGATTGGCTTATTAAAAAAGCCA
AGGCTGCCATTGATGAACTCGCGGAGCTTCCGGCATGGAAGCCCACTGCCACTACGGCGACG
ACAACCCCAAATTCAACATCGATTGCAGATTTTGAGCAAAACCCAGATCAACAAAACTCAAA
TCATCATCAACTAAGTCATTTCGAACAACACCCAGATGATAGTATTGTTGATAATCAAATGGGT
AATTCACAAAACTCAAGCTTCTTGCCTCCGTCTCTTGATTCCGACTCTATAGCTGATACAATCA
AGTCATTTTTTCCGATGGGTGCTTCAACAAATCCAGGAAATAATACTTCTTCAGGTATGCAATT
TCATCAAAGTTTTCCACCTCAGGATTTGCTTTCAAGAACCAGTAGTCGAAGTCAAGATCTGAG
GCTTTCTCTTCAATCATTCCAAGATCCGATTCTCCAGAACCACCACCATCACCACCAACAAAA
CGAACAGGGTAATAATAACAATAGCAATATATTCTTTGACGGGTCGGGTTGGTCCGACAACCA
GCCTGGTGGGTTTCAAAGAATGGTGGCGTGGGGTGGTGTAGGAGGCGGAGACGCTGTTTCCG
CCGGATTTGTCTTCAGTTCGCAGCCGTCGCCGTCGACGCCTTTTCTGCAACCGTTGTTCGGTC
AAACAACAAACAATCAGTTATTCAACAATTCTCAGAGGGGACCCCTTCAGTCCAGTAACGCA
CCTTCGTTTCGTGCTTGGATCGACCCGCCTCCACCGTTCACCGGTGTCGCCATCGATCAACAC
CCAACCCTAGCTTTCCATCACCCATCTTCCATGTCCGGTTTCGCCTCCGGTTTAGGTGGGTTTT
CCGGGTTTCGTATTCCAGCACGAATTCAAGGTGAAGAGGAGGAACACGACGGCATCTCCGAT
AAGCCGTCCTCTGCATCCTCCGATTCTCGCCATTGAGTAAAAGAAACCCATCACCTGTTTCTC
AATTCCGCCTGATTTCGTTTTATTCTTAATTTCATCAATAATTACAGATCATCTTCATCCTCCATA
ATCCCTCTCAAACAGGAAAATTTACGTCAATCAATGTACTTCAAAGCAAAGAGAGGAGGGGA
GAAGCTTCAATGGCTGCTACACAGTGGGAAAAAG**AAACCGAATCCATTCACAGG**TTCTTCA
GTCATCACTGGTGTTTTTACGATTTAGCTTTCATGTTTGAATAGAGCTCTTTGTTTAGCTCTAAA
CCCTGTTTTAAATTATCTGGATTTAATGTTTTTAGACCATGTATGTGACTGTGTGTTATGAACTC
AGATTCTCTCAAGGATAAATTAAAACCACAACTGATGGATTCACACTTCTTCTGGGATTAATGA
ACGAAAACAGAGATTACACAAGAAATTGATAAGAAAAAGAATGAAATAGAAATAGCAATTCG
AGCTGCTACGGACTCCCAGAGAAGTAGAAACTTCTAATATTCTTCCATGCCATCACCAGGCTC
CCTACCCATCCTTATAACGGAATGTCAGTATTTCTCTAAATTCCCACTTTGCCCCTGCTCCATGT
CAGCAACATCCTCCTGGGTGATAGTGGTCCCTTGTCCTTTTAGAATCTTTTTGTGTTTCTTTCTT

コメント [S1]: Primer sequence LG5_v8_252.743Mbp_Salinas_F

TGGTACGTGAAATGTAGTGGTGGGTGCACCACATTACCCTGCCCCCAAAGCAAGACCTTGTCC
TCAAGGTCAAAGTCTGGAAAGGTGCCTTGAATAGTTGTGAAATCTTCCCAGGTGGAATCAAA
ATCAGGAACCCCCTTCCATTTGATCAACACTTCTGTTCTTCGACCTGTGTCTTCTTGCTTGGTA
CGAACCCCCAGCAATGCTTCTGGTTCCCAGTCAAGACGCATGTCTTCCATTAGGTGGGTTGGC
AATTTCGGGTATGCTGACTGATTGCCTATTGCTTTCTTGAGTTGCGAAACATGAAAGACCGGAT
GGATCCTAGCATCCTCTGGTAGCTGTAAGTGATAAGCAACCGCACCAATTTTTTTGATAATCAC
GAATGGTCCATAAAATCGAGCCGCCAGCTTCTGGAAAGGTCTATTTGTCACTGACCTTTGTCTA
TACGGTTGCAATTTTAGATAGACCCACTCTCCTTCTTCATATGACACATCTTTTCTACCCTTATC
AGCATCTGTCTTCATTTTTTGTTGAGCCTGAAGCAAGTGGCCCTTAAGATCATCCAACGTGGC
GTCTCTTTCCATCAGTTGTTCTTCCACAGCCAAAACTCCTGTTTGCCCTTGCTGAACCCGTATA
ACACGCGGCGGGTCACGCCCATACAAGGCCTTAAAAGGCGTGATTTTGCTGGCGGTGTGATA
AGCGGTGTTGTGCCAAAACTCTGCCCATGGTAACCATTGCGACCAGGAACGAGGCTTACCGT
GGATGAAACACCTTAAATACATCTCCACCGACTTGTTGACGATCTCCGTTTGTCCATCGGTTTG
CGGGTGGTAGGCTGTGCTTCTTAATAACTGAGTCCCTTGTAATCGAAACAATTCACGCCAAAA
CAAACTCATAAACACCTTGTCTCGATCAGAGACTATGGAAGATGGAAACCCATGAAGACGAA
CTACTTCCTTAATGAAAATTGCTGCAATTGTTGTTGCTGAGAAGGGGTGTTTAACCGCTATGAA
ATGGGAGTACTTAGTAAGGCGATCAACTACAACCAATATGGCATTGTAACCATGCGAGTTAGG
TAGGCCTTCGATAAAATCCATGGATATCTCATCCCAAACCTGGCTCGGAAGGGGTAATGGTTGT
AATAACCCCGCCGGATGAGTAGTTAAAGCTTTTTGGGTTTGGCACACGACACAGGCTTGTACA
TGTTTCTGGACACTCTTCCTCATTCCTACCCAATACCACTCCGCCGCCAACCTTTGGTAAGTTC
TTAATTCCCCTGCATGTCCTCCCATCACCGAATCATGGAATTCCTTCAATAGCTTGGGAATCAA
CTCCGATTTTTGAGGTATGACCAACCTACCTTTATACATCAATTTCCCACCTTCCATTGTGTAGC
CTTTGTGGTGTTTTCCTTCCTCACATATGTCTTTTTTTACCTGCTGAATGAAGGAATCTTCAGCT
ATTTCTTTATCCAATTCTCCCAACGGGAAGGTCCAAGTAGATGTAAGCATGTTGATTTCGGTTG
ATTCGGAGAGTTCTCGAGAGAGAGCATCTGCCACCTTATTGGTAGCCCCCGGTTTGTACTGAA
TCTCAAAGTCGAAGCCCAATAATTTATACATCCATTTTTGGTATTCTGGGCCCACTTCTCGTTGT
TCCATCAAATATTTGAGACTGCGTTGGTCTGTTCTAATTAAAAAATGGCGTCCCATCAAGTAAT
GTCTCCACCTTTTAACAGCCAAAACAATGGCCATCAATTCCTTTTCATAAATCGATTTAAGCCT
ATTCTTTACCCCTAAAACTTGGCTATAAAAAGCCACTGGATGCTTGTTTTGCAGCAGGACTGCT
CCTATCCCTTTTCCGGAAGCATCCGTTTCAATGATAAACAATTTGGTAAAGTCCGGCATGGCCA
ATATTGGTGCTTTCATTAGGGCTGCCTTTAGAGTGTTGAAAGCTTGGATGGCGATTGGTGACCA
CCCAAAGCAATCCTTGCGTAATTGATCCGTCAGAGGTGCTGCAATACTCGCATACCCTGAAAT
AAACTTTCTGTAATATCCTGTGAGACCCAGGAACCCCCTGAGTTCACGCAAAGATTTTGGAAT
GGGCCATTGTTCAATTGCTTTTACCTTTTCAGGGTCGACTGCTACCCCTTGTTTTGAAATAACA
TGGCCTAAGTAGGCTATTTGGTTTTTACCCCATTCACACTTGGCTGAATTAGCATATAACTCGTG
TTGCTTCAATGTTTCCAAAACAATCTTCAAATGGGACAAATGTTGACTTTCGTCTCGGCTATAA

ATTAGGATGTCGTCAAAGAATACCAGCACAAACTTTCGTAGATGAGGTCTGAAAATCTCATTC
ATGACAGATTGAAAGGTGGCTGGGGCATTAGTAAGGCCGAAGGGCATAACCTTGAACTCATAA
TGCCCTTCATGCGTTCTAAACGCTGTTTTATGAATATCGTCCACCTTCATACGGATTTGATGATA
ACCTGATTTCAAATCCAGCTTGGAAAACATCCGTGATCCGTGTAATTCATCTAAAAGCTCGTCT
ATCACGGGTATGGGAAACTTGTCTTTAACCGTCACCTTGTTAAGGGCCCGATAGTCAACACAG
AACCTCCACGAGCCATCTTTTTTTTAACCAACAACACGGGGCTAGCAAAAGGACTTCGGGA
CGGTTGGATGATTTTGGAGTCGAGCATGTCGTGAAGCAATCTTTCAATTTCTGCTTTTTGGGTA
TGAGAGTACCTATAAGGACGTACCGTCACCGGTCCAGTTCCCTCCTTCAGATTAATGGCGTGC
TGGTGATTCCTTAGTGGGGGTAGGCCTCCTTTCCAAGAAAAAACAGAAGCATACCTCTGTAAT
ACTGGAAGTAGAAAGGGAGGGCAGTCTTTAATGTGATTCACGGGCTGTTGGTCCTCCACTTG
GTTCAACTGGACCCAATATCCACCTCCCACAGAGCCAATGGTTCTAATCATGGTTTTTAAGGA
AATTTTTGCCCTTTCTAAGGATGGGTCTCCCCGGAGAGTCACCCCGTGGCCTTCTATTTGAAAC
TTCATGACTTGCGTTTTCCAATTAGTAGTCACCGCCCCTAACTTCTCCAACCATTGGATTCCCA
GAATTACGTCTGAATTTCCCAAGGTGAGCGGTAAGAAATCCTCTCTAATATCCAGCCCTTGAAT
ATGTATTAATACCCCTTGGCAATTTCCTCTTCCGGTCACTGATTCTCCTGTGCCCAAAGAAACC
CCAAACCCCGGAGTAGGGTTGACTGGAATCTGTAATTGATCTATAGTTGCCAAGGAAAGGAAA
TTGTGAGTTGCTCCCGGATCAATGAGTACCACCACTTCTGTGTCCTTAATGATTCCCTTAAGTT
TGAGGGTCTTAGGATTGAGGTTACCAGTAACTGAATTCAAACAGACACCTGATATCAATGGGA
GTTCGTTAGTGTCCAAATTTGCAAATTCATTGACTTCGTCTGGTGGGTCTTCTTCCCCTGTTTC
TTCTTCTTGCATTAAGAGTACACTGAGTTCCTTCCTCTTGCATTGATGCCCTGCACTCCATTTCT
CATCACAACGAAAGCAAAGCCCTTTTGATCGTTTATATTGCAGTTCTTTATCCGACAATCTTCT
CACTTCCCCGCCTGGTTTGCTGGGGTTCTTAATCGACACGGCTGGGGTGTGGGTTGGTACCGT
AGACGATCGAGGGGGGAAATAAGTGGTTTTGAGCGGGGTTATCAGGGGTGTTCCAGAAAGAG
TACCTGATGTGTTAGTCCGAGGAGTTACAGTGGAGAGAGAGGATTTCCGATGGAGTTGGGAA
TTAAGCTTGCGTTCGACTTTGATGGCCAACGTCATAGCTTGTTCCACTGATATGGGGCTCAATA
ACTGGACCTCAGACCTAATTTCCTCCTGTAGCCCGTTGATGAAGTGGCCCAAGGTGATGTCAT
CAGGTATGTTGTTTAATGGGGCAAGTAGTTCAATAAAGGCTCGTTGGTATTCTAAAACCGACC
CAGATTGAATTAGGGCTAACCACTGCTGGTGTAACGTACCTGCAGTAACCGGACGAAACTGTC
GTAATAGGAGAGATTTCATCTCATCCCACCGTGTCACCGGCCGGCGAGTGTGCTCCCACTGGT
ACCAGAGAAGGGCGTCTCCTTCAAGAGCCACCACCGCTGCCTCCATTTTATCAGCTTCTGATA
ATCTGTAGAAATTGAAATAGCGTTCTGCACGTAATATCCATCCATCGGGGTTTTCCCCGTCGAA
GAGGGGCATATCGAGTTTCCGAAATCGCCAATTGGTCCCTCCTGTTGGTTCACCGTTATTGGTT
CGGCCGTTGCCCCCATCGCCACTACCTCCCGACCCACCTACGACGCCGCTGCGGTATCCCCCA
GCGGATTCTTTTGGTGTCGCCAAAATGGAAGAAACAGGTTCGGACTCCTTCTCCCCTTCTCCT
GACTTCATCCCTTTAGTTACAGCTCTCAAAACCTCTTCCAACTTCTGGTCCATCTTAAGCTGAT
TTTGGTCCATCTTCAGCTGGATGGAAG**CTATGGAGTCCGCCTGATGT**TGGATCTGATTACCT

コメント [S2]: Primer sequence LG5_v8_252.743Mbp_Empire_F

TGGGTCTCTAAGCGATCATACAACACACCTAATTCTGACTCCAGCGATTCAACCCTCTTGAGTT
GAGCTCCTCCAGCCTCTTTCCCACCTCCCTTTTCTCCAGCCATAATCCCAGGTGGTTGGTTGCT
CTGATACCAATTTGTTATGAACTCAGATTCTCTCAAGATAAATTAAAACCACAACTGATGGATT
CACACTTCTTCTGGGATTAATGAACGAAAACAGAGATTACACAAGAAATTGATAAGAAAAAG
AATGAAATAGAAATAGCAATTCGAGCTGCTACGGACTCCCAGAGAAGTAGAAACTTCTAATAT
TCTTCCATGCCATCACCAGGCTCCCTACCCATCCTTATAACGGAATGTCAGTATTTCTCTAAATT
CCCACTTTGCCCCTGCTCCATGTCAGCAACATCCTCCTGGGTGATAGTGGTCCCTTGTCCTTTT
AGAATCTTTTTGTGTTTCTTTCTTTGGTACGTGAAATGTAGTGGTGGGTGCACCACACTGTGCC
AATTGCCTTCTTTTGCTATATAATTATCAAAAATACTCTGCTAAAAACAGAGCAAGTGATTTGTT
TTGGTTATAATTTCACTGTTATTCGCCCAACTTTAGTAAAACAAGAATCTTTTGAATATCAACCA
AATCAACACATTCTTTGTGTGTGATTGATTGAAATTTGAAAGGCCATGA**GATGAGAGGAGCA**
AATGGGATGTTTCCCTTTGTTTAGAAAATTGTCAATTTTTTATGTATGTGGGGTTTGAAAGAGA
GAGCTCCAATTTTTGTCTCTCTTTTTACCCAATTCTTGCAATCTTGCTTCATATGCTTTTTCATTA
GTGTGCCCTAATGGAACATGATGAAGAAAGGAAAGGGTAG

コメント [S3]: LG5_v8_252.743Mbp_R

LsTCP4 CDS

LsTCP4 3’UTR

LsTCP4 intron

Ty3/gypsy retrotransposon-like insertion

3’flanking sequence of *LsTCP4*

Fig. S5.

Sequence of *LsTCP4*, Ty3/gypsy retrotransposon like insert and position of LG5_v8_252.743Mbp primers.

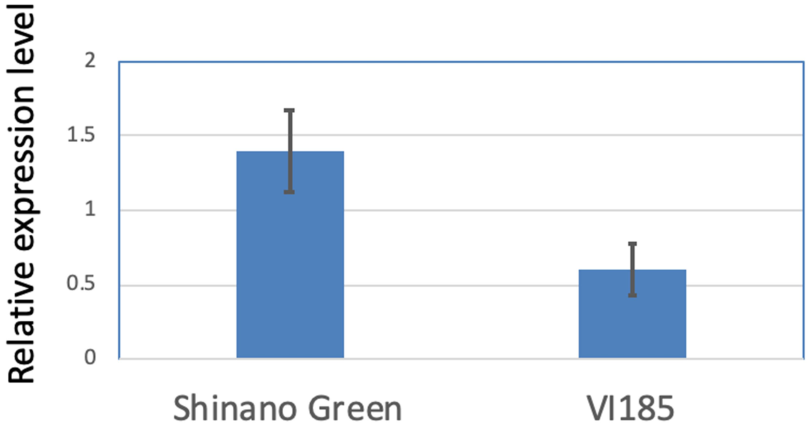

Fig. S6

Comparison of *LsTCP4* expression in the leaves between ShinanoGreen and VI185 by quantitative RT-PCR. Quantitative RT-PCR analysis of *LsTCP4* gene was carried out in each three replicated plants of ShinanoGreen and VI185. *LsTCP4* expression level was normalized by the expression of *TUB* gene in each sample. Normalized expression value in one replicate of ShinanoGreen was defined as the standard (=1) and relative expression level in other samples were calculated. Averaged relative expression level and standard error among triplicates were indicated.
