# Supplementary material for: A CIN-like TCP transcription factor (LsTCP4) having retrotransposon insertion associates with a shift from Salinas type to Empire type in crisphead lettuce (Lactuca sativa L.)
Source: Hortic Res. 2020 Feb 1;7:15. doi: 10.1038/s41438-020-0241-4 (PMC6994696; doi:10.1038/s41438-020-0241-4)
Supplement: Supplementary file 5 — Supplementary Tables [file 41438_2020_241_MOESM5_ESM.pdf]

Supplementary Information

A CIN-like TCP transcription factor (*LsTCP4*) having retrotransposon insertion associates wit[illegible]

This file includes:

Supplementary Table S1 to S7

Table S1 The mean values of inner stem length for 10 cultivars in field cultivation tests.

Year	Empire type										Salinas type									
	SummerAce		ShinanoPower		Patriot		Olympia		ShinanoHope		Lushina66		Mayer		Raptor		Vlettuce		ShinanoGreen	
	Average (cm)	SD	Average (cm)	SD	Average (cm)	SD	Average (cm)	SD	Average (cm)	SD	Average (cm)	SD	Average (cm)	SD	Average (cm)	SD	Average (cm)	SD	Average (cm)	SD
2013	5.00	±0.46 a	5.37	±0.42 ab	5.95	±0.61 ab	6.88	±0.56 ab	6.93	±0.83 ab	4.97	±0.30 a	5.72	±0.34 ab	7.52	±0.59 ab	7.93	±1.20 b	11.97	±3.36 c
2014	3.92	±0.26 a	3.27	±0.51 a	4.78	±0.37 ab	5.15	±0.37 ab	6.05	±0.85 bc	3.42	±0.34 a	4.77	±0.94 ab	5.27	±0.62 ab	7.37	±1.05 c	10.13	±2.19 d
2015	4.23	±0.91 a	4.95	±0.65 a	5.68	±0.76 a	5.82	±0.43 a	7.20	±1.70 a	3.70	±0.67 a	5.22	±0.93 a	6.93	±1.66 a	13.63	±6.18 b	18.53	±1.79 b
2016	7.92	±1.35 ab	6.42	±0.44 a	8.78	±1.56 ab	10.23	±2.90 ab	10.32	±1.43 ab	6.30	±0.69 a	6.92	±1.05 ab	10.33	±1.37 ab	11.37	±1.33 b	16.25	±4.51 c
2018	5.53	±0.44 a	5.43	±0.45 a	5.83	±0.47 a	9.27	±1.78 a	6.13	±0.62 a	5.32	±0.58 a	7.15	±0.77 a	8.48	±1.30 a	15.85	±3.71 b	21.10	±3.39 c
Total	5.32	±1.62 ac	5.09	±1.15 ab	6.21	±1.59 ac	7.47	±2.47 bc	7.33	±1.92 bc	4.74	±1.19 a	5.95	±1.23 ac	7.71	±2.03 c	11.23	±4.55 d	15.60	±5.09 e

Table S2 Tipburn incidence for 10 cultivars in field cultivation tests.

Year	Cultivar	Type	Outside Tipburn			Inside Tipburn		
			%Tipburn	No.with tipburn	No. no tipburn	%Tipburn	No.with tipburn	No. no tipburn
2013	Olympia	Empire	26.7	8	22	40	4	6
2014	Olympia	Empire	13.3	4	26	70	7	3
2015	Olympia	Empire	26.7	8	22	100	10	0
2016	Olympia	Empire	6.7	2	28	100	10	0
2018	Olympia	Empire	46.7	14	16	70	7	3
2013	Patriot	Empire	20.0	6	24	50	5	5
2014	Patriot	Empire	0.0	0	30	70	7	3
2015	Patriot	Empire	0.0	0	30	100	10	0
2016	Patriot	Empire	6.7	2	28	100	10	0
2018	Patriot	Empire	70.0	21	9	70	7	3
2013	ShinanoHope	Empire	26.7	8	22	100	10	0
2014	ShinanoHope	Empire	10.0	3	27	100	10	0
2015	ShinanoHope	Empire	30.0	9	21	100	10	0
2016	ShinanoHope	Empire	16.7	5	25	100	10	0
2018	ShinanoHope	Empire	60.0	18	12	80	8	2
2013	ShinanoPower	Empire	66.7	20	10	100	10	0
2014	ShinanoPower	Empire	16.7	5	25	80	8	2
2015	ShinanoPower	Empire	66.7	20	10	100	10	0
2016	ShinanoPower	Empire	60.0	18	12	100	10	0
2018	ShinanoPower	Empire	80.0	24	6	100	10	0
2013	SummerAce	Empire	30.0	9	21	90	9	1
2014	SummerAce	Empire	3.3	1	29	70	7	3
2015	SummerAce	Empire	10.0	3	27	100	10	0
2016	SummerAce	Empire	10.0	3	27	100	10	0
2018	SummerAce	Empire	40.0	12	18	100	10	0
2013	Lushina66	Salinas	3.3	1	29	30	3	7
2014	Lushina66	Salinas	16.7	5	25	50	5	5
2015	Lushina66	Salinas	0.0	0	30	30	3	7
2016	Lushina66	Salinas	0.0	0	30	40	4	6
2018	Lushina66	Salinas	3.3	1	29	0	0	10
2013	Mayer	Salinas	13.3	4	26	50	5	5
2014	Mayer	Salinas	0.0	0	30	20	2	8
2015	Mayer	Salinas	0.0	0	30	40	4	6
2016	Mayer	Salinas	3.3	1	29	100	10	0
2018	Mayer	Salinas	10.0	3	27	50	5	5
2013	Raptor	Salinas	53.3	16	14	40	4	6
2014	Raptor	Salinas	6.7	2	28	40	4	6
2015	Raptor	Salinas	0.0	0	30	90	9	1
2016	Raptor	Salinas	10.0	3	27	100	10	0
2018	Raptor	Salinas	30.0	9	21	100	10	0
2013	ShinanoGreen	Salinas	0.0	0	30	0	0	10
2014	ShinanoGreen	Salinas	0.0	0	30	20	2	8
2015	ShinanoGreen	Salinas	0.0	0	30	0	0	10
2016	ShinanoGreen	Salinas	0.0	0	30	40	4	6
2018	ShinanoGreen	Salinas	10.0	3	27	10	1	9
2013	Vlettuce	Salinas	3.3	1	29	0	0	10
2014	Vlettuce	Salinas	3.3	1	29	30	3	7
2015	Vlettuce	Salinas	0.0	0	30	70	7	3
2016	Vlettuce	Salinas	13.3	4	26	90	9	1
2018	Vlettuce	Salinas	6.7	2	28	0	0	10

Table S3 Summary of integrated lettuce linkage groups.

Linkage groups	Total mapped tags	Common tags		ShinanoGreen unique tags		VI185 unique tags		Linkage construct marker		
								No.biallelic tags	Map length	Average interval between markers
		No.RAD-tags	(%)	No.RAD-tags	(%)	No.RAD-tags	(%)		(cM)	(cM)
LG1	42512	21914	51.5	10990	25.9	9608	22.6	682	153.9	0.2
LG2	40733	22562	55.4	8516	20.9	9655	23.7	588	142.9	0.2
LG3	52007	31542	60.6	10255	19.7	10210	19.6	504	199.7	0.4
LG4	70863	47816	67.5	10174	14.4	12873	18.2	140	213.8	1.5
LG5	66541	38787	58.3	13678	20.6	14076	21.2	810	153.1	0.2
LG6	36722	23481	63.9	6024	16.4	7217	19.7	227	157.5	0.7
LG7	37803	23138	61.2	6878	18.2	7787	20.6	306	134.8	0.4
LG8	61349	36976	60.3	11460	18.7	12913	21.0	644	195.8	0.3
LG9	40914	23355	57.1	8439	20.6	9120	22.3	616	177.7	0.3
Total	449444	269571	60.0	86414	19.2	93459	20.8	4517	1529.2	0.3

Table S4 Developed markers in LG5 based on PCR to distinguish between VI185/ShinanoGreen.

Primer name	Marker type	Primer sequence (5'-3')	PCR product size	
			Empire type	Salinas Type
LG5_v8_251.667Mb_F<br>LG5_v8_251.667Mbp_R	Indel	CGTTTATGTATCGGGGGAGA<br>GGAAATGGAGGAACGGAGTT	194bp	212bp
LG5_v8_251.738Mbp_F<br>LG5_v8_251.738Mbp_R	Indel	CCTTTTTCCCTCTTTTCTTTCC<br>CCAAACGCTGTTTTGCTGTA	236bp	209bp
LG5_v8_252.704Mbp_DdeI_F<br>LG5_v8_252.704Mbp_DdeI_R	CAPs (DdeI cut)	GCCAAGGTTTTCGTTGACAT<br>TCCGGTTCGTGTCTGTGTAA	380bp	243bp<br>137bp
LG5_v8_252.743Mbp_Salinas_F<br>LG5_v8_252.743Mbp_Empire_F<br>LG5_v8_252.743Mbp_R	Indel	AAACCGAATCCATTCACAGG<br>CTATGGAGTCCGCCTGATGT<br>TCCCATTTGCTCCTCTCATC	744bp	346bp
LG5_v8_252.927Mbp_F<br>LG5_v8_252.927Mbp_R	Indel	TTGTTGTTGTTGTTGTTGACAGA<br>GCGGGATTGGAAAGAGAGAT	196bp	181bp
LG5_v8_252.999Mbp_F<br>LG5_v8_252.999Mbp_R	Indel	CACAAACCGCACTGCATAAT<br>TTTCTGTGCCCATTCCAGTT	202bp	212bp

Table S5 TCP-like gene in *L.sativa*

						Total reads									The RPKM								
Name	Chromosome	miR319 target site	BLAST TOP Hit in Arabidopsis (AGI)	BLAST TOP Hit in Arabidopsis (Gene Name)	TCP type	*ShinanoGreen/VI185*	*ShinanoGreen*	*VI185*	*ShinanoGreen_1*	*ShinanoGreen_2*	*ShinanoGreen_3*	*VI185_1*	*VI185_2*	*VI185_3*	*ShinanoGreen/VI185*	*ShinanoGreen*	*VI185*	*ShinanoGreen_1*	*ShinanoGreen_2*	*ShinanoGreen_3*	*VI185_1*	*VI185_2*	*VI185_3*
Lsat_1_v5_gn_1_115940	LG1		AT3G15030.3	TCP4	CIN		0	0	0	0	0	0	0	0		0	0	0	0	0	0	0	0
Lsat_1_v5_gn_1_115960	LG1		AT3G15030.3	TCP4	CIN		0	0	0	0	0	0	0	0		0	0	0	0	0	0	0	0
Lsat_1_v5_gn_1_120721	LG1		AT2G31070.1	TCP10	CIN		0	0	0	0	0	0	0	0		0	0	0	0	0	0	0	0
Lsat_1_v5_gn_1_23301	LG1		AT3G15030.3	TCP4	CIN		0	0	0	0	0	0	0	0		0	0	0	0	0	0	0	0
Lsat_1_v5_gn_3_73860	LG3		AT3G15030.3	TCP4	CIN		0	0	0	0	0	0	0	0		0	0	0	0	0	0	0	0
Lsat_1_v5_gn_4_110881	LG4		AT5G60970.1	TCP5	CIN	1.5	56.7	37.3	49	60	61	25	38	49	1.5	1.3	0.9	0.9	1.5	1.6	0.6	0.9	1.2
Lsat_1_v5_gn_4_29640	LG4		AT4G18390.2	TCP2	CIN	1.3	1081.7	862.7	1257	1007	981	803	846	939	1.2	32.6	27.4	31.9	32.9	33.0	26.0	27.1	29.1
Lsat_1_v5_gn_5_127021 (LsTCP4)	LG5	●	AT3G15030.3	TCP4	CIN	1.7	1624.0	932.3	1914	1514	1444	834	964	999	1.7	57.4	34.8	57.0	58.1	57.1	31.7	36.3	36.3
Lsat_1_v5_gn_5_127080	LG5		AT2G31070.1	TCP10	CIN		0	0	0	0	0	0	0	0		0	0	0	0	0	0	0	0
Lsat_1_v5_gn_5_22620	LG5		AT5G60970.1	TCP5	CIN		0.3	0	1	0	0	0	0	0		0.0	0	0.0	0	0	0	0	0
Lsat_1_v5_gn_7_23540	LG7		AT2G31070.1	TCP10	CIN	0.6	17.3	28.3	22	10	20	39	17	29	0.6	0.8	1.4	0.9	0.5	1.0	2.0	0.9	1.4
Lsat_1_v5_gn_7_27781	LG7		AT4G18390.2	TCP2	CIN	1.2	1562.3	1282.3	1979	1362	1346	1201	1230	1416	1.1	16.3	14.2	17.6	15.6	15.9	13.6	13.8	15.3
Lsat_1_v5_gn_9_99020	LG9	●	AT3G15030.3	TCP4	CIN	1.0	180	173	181	99	260	177	151	191	1.0	3.3	3.3	2.7	1.9	5.2	3.4	2.9	3.5
Lsat_1_v5_gn_1_16061	LG1		AT3G18550.2	BRC1, TCP18	CYC/TB1	0	0	1	0	0	0	3	0	0	0	0	0.0	0	0	0	0.1	0	0
Lsat_1_v5_gn_1_4641	LG1		AT1G67260.1	TCP1	CYC/TB1		0	0	0	0	0	0	0	0		0	0	0	0	0	0	0	0
Lsat_1_v5_gn_3_94461	LG3		AT1G68800.1	BRC2, TCP12	CYC/TB1		0	0	0	0	0	0	0	0		0	0	0	0	0	0	0	0
Lsat_1_v5_gn_4_112280	LG4		AT3G18550.1	BRC1, TCP18	CYC/TB1	0	0	0.3	0	0	0	0	1	0	0	0	0.0	0	0	0	0	0.0	0
Lsat_1_v5_gn_4_175901	LG4		AT1G67260.2	TCP1	CYC/TB1		0	0	0	0	0	0	0	0		0	0	0	0	0	0	0	0
Lsat_1_v5_gn_4_175961	LG4		AT1G67260.2	TCP1	CYC/TB1		0	0	0	0	0	0	0	0		0	0	0	0	0	0	0	0
Lsat_1_v5_gn_4_19160	LG4		AT1G67260.2	TCP1	CYC/TB1	0	0	1.3	0	0	0	1	1	2	0	0	0.0	0	0	0	0.0	0.0	0.1
Lsat_1_v5_gn_8_122100	LG8		AT1G68800.1	BRC2, TCP12	CYC/TB1	2	0.7	0.3	1	1	0	0	1	0	1.8	0.0	0.0	0.0	0.1	0	0	0.1	0
Lsat_1_v5_gn_8_37420	LG8		AT1G67260.2	TCP1	CYC/TB1	0.4	1.0	2.7	0	0	3	3	1	4	0.4	0.0	0.1	0	0	0.1	0.1	0.0	0.1
Lsat_1_v5_gn_1_13200	LG1		AT3G47620.1	TCP14, TCP14	PCF	1.5	184.3	121.0	272	123	158	142	101	120	1.4	4.7	3.3	6.0	3.5	4.6	4.0	2.8	3.2
Lsat_1_v5_gn_1_52001	LG1		AT1G58100.1	TCP8	PCF		0	0	0	0	0	0	0	0		0	0	0	0	0	0	0	0
Lsat_1_v5_gn_2_130241	LG2		AT5G51910.2	TCP19	PCF	1.3	30.0	22.7	43	35	12	29	24	15	1.2	1.0	0.8	1.3	1.3	0.5	1.1	0.9	0.5
Lsat_1_v5_gn_2_94320	LG2		AT3G27010.1	PCF1, TCP20	PCF	1.3	187.7	146.0	283	165	115	167	142	129	1.2	5.1	4.3	6.6	5.0	3.6	5.0	4.2	3.7
Lsat_1_v5_gn_2_99740	LG2		AT2G45680.1	TCP9	PCF		0	0	0	0	0	0	0	0		0	0	0	0	0	0	0	0
Lsat_1_v5_gn_3_36601	LG3		AT5G23280.1	TCP7	PCF	1.2	800.0	648.3	976	686	738	608	566	771	1.2	21.6	18.5	22.3	20.2	22.4	17.7	16.4	21.5
Lsat_1_v5_gn_3_52660	LG3		AT1G69690.1	TCP15	PCF	0.9	6.0	7.0	10	0	8	13	2	6	0.8	0.2	0.2	0.3	0	0.3	0.4	0.1	0.2
Lsat_1_v5_gn_4_107781	LG4		AT3G47620.1	TCP14	PCF	0.9	670.3	727.3	994	486	531	801	692	689	0.8	16.2	19.1	20.8	13.1	14.7	21.4	18.3	17.6
Lsat_1_v5_gn_4_64660	LG4		AT5G23280.1	TCP7	PCF	0.8	324.7	412.0	441	207	326	425	414	397	0.7	10.0	13.6	11.6	7.0	11.4	14.3	13.8	12.7
Lsat_1_v5_gn_5_103680	LG5		AT1G58100.1	TCP8	PCF	0.8	646.7	820.7	925	505	510	864	780	818	0.7	12.5	17.1	15.4	10.8	11.2	18.3	16.4	16.6
Lsat_1_v5_gn_5_19421	LG5		AT3G47620.1	TCP14	PCF	1.1	113.3	100.3	175	74	91	116	95	90	1.0	3.0	2.9	4.0	2.2	2.8	3.4	2.7	2.5
Lsat_1_v5_gn_6_45361	LG6		AT5G23280.1	TCP7	PCF	1.5	39.3	26.7	60	35	23	33	26	21	1.3	2.0	1.5	2.6	2.0	1.3	1.8	1.4	1.1
Lsat_1_v5_gn_7_6960	LG7		AT5G51910.2	TCP19	PCF	1.2	29.3	24.0	33	30	25	33	20	19	1.2	0.7	0.6	0.7	0.8	0.7	0.8	0.5	0.5
Lsat_1_v5_gn_8_51760	LG8		AT1G69690.1	TCP15	PCF	3.4	35.0	10.3	46	25	34	9	8	14	3.2	1.1	0.3	1.2	0.8	1.2	0.3	0.3	0.4
Lsat_1_v5_gn_8_7700	LG8		AT5G51910.2	TCP19	PCF	1.2	16.7	14.3	21	14	15	10	18	15	1.1	0.5	0.5	0.6	0.5	0.5	0.3	0.6	0.5
Lsat_1_v5_gn_9_53821	LG9		AT5G51910.2	TCP19	PCF	1.0	264.3	276.0	526	117	150	348	229	251	0.8	5.3	6.2	9.5	2.7	3.6	8.0	5.2	5.5

Table S6 Meteorological conditions of field cultivation tests.

Year	Planting	Harvest survey	Cultivation period (Day)	Total precipitation (mm/m^2)	Temperature Highest (℃)	Temperature Mean (℃)	Temperature Lowest (℃)
2013	21-Jun	1-Aug	41	214.5	35	22.7	12.9
2014	9-Jun	22-Jul	43	139	31.3	20.8	9.9
2015	16-Jun	30-Jul	44	203.5	34.5	21.9	12.7
2016	16-Jun	28-Jul	42	126	33	22.3	11.8
2018	29-Jun	6-Aug	38	38.5	36.7	25.9	17.9

Table S7 Number of mapped reads in RNA-Seq analysis

	Reads mapped in pairs	Reads mapped in broken pairs	Reads not mapped	Total reads used for mapping (Q30)
ShinanoGreen_1	56,988,088	6,293,055	1,910,007	65,191,150
ShinanoGreen_2	44,254,928	5,190,976	1,456,048	50,901,952
ShinanoGreen_3	42,965,776	4,884,985	1,566,433	49,417,194
VI185_1	44,688,454	5,119,768	1,507,316	51,315,538
VI185_2	45,094,752	4,795,157	1,522,819	51,412,728
VI185_3	46,718,338	6,844,921	1,617,157	55,180,416
